# Genome-wide identification and expression characterization of ABCC-MRP transporters in hexaploid wheat

**DOI:** 10.3389/fpls.2015.00488

**Published:** 2015-07-01

**Authors:** Kaushal K. Bhati, Shivani Sharma, Sipla Aggarwal, Mandeep Kaur, Vishnu Shukla, Jagdeep Kaur, Shrikant Mantri, Ajay K. Pandey

**Affiliations:** ^1^Department of Biotechnology, National Agri-Food Biotechnology InstitutePunjab, India; ^2^Department of Biotechnology, Panjab UniversityPunjab, India

**Keywords:** ABCC-MRP proteins, cadmium stress, *Triticum aestivum*, detoxification, yeast complementation, seed development

## Abstract

The ABCC multidrug resistance associated proteins (ABCC-MRP), a subclass of ABC transporters are involved in multiple physiological processes that include cellular homeostasis, metal detoxification, and transport of glutathione-conjugates. Although they are well-studied in humans, yeast, and *Arabidopsis*, limited efforts have been made to address their possible role in crop like wheat. In the present work, 18 wheat ABCC-MRP proteins were identified that showed the uniform distribution with sub-families from rice and *Arabidopsis*. Organ-specific quantitative expression analysis of wheat ABCC genes indicated significantly higher accumulation in roots (*TaABCC2, TaABCC3*, and *TaABCC11* and *TaABCC12*), stem (*TaABCC1*), leaves (*TaABCC16 and TaABCC17*), flag leaf (*TaABCC14* and *TaABCC15*), and seeds (*TaABCC6*, *TaABCC8*, *TaABCC12, TaABCC13*, and *TaABCC17*) implicating their role in the respective tissues. Differential transcript expression patterns were observed for *TaABCC* genes during grain maturation speculating their role during seed development. Hormone treatment experiments indicated that some of the *ABCC* genes could be transcriptionally regulated during seed development. In the presence of Cd or hydrogen peroxide, distinct molecular expression of wheat *ABCC* genes was observed in the wheat seedlings, suggesting their possible role during heavy metal generated oxidative stress. Functional characterization of the wheat transporter, TaABCC13 a homolog of maize *LPA1* confirms its role in glutathione-mediated detoxification pathway and is able to utilize adenine biosynthetic intermediates as a substrate. This is the first comprehensive inventory of wheat ABCC-MRP gene subfamily.

## Introduction

ATP-binding cassette transporter (ABC) proteins are found in all living organisms and constitute one of the largest known superfamily (Henikoff et al., 1997; Rea, 1999). They are involved in multitude functions in animals and plants for the transport of broad substrates ranging from glutathione-conjugates, xenobiotic compounds, intermediate metabolites and hormones (Rea, 1999; Bakos et al., 2000; Augustine et al., 2005; Kang et al., 2010; Klaassen and Aleksunes, 2010). The ABCC subfamily of these transporters classically referred as multi drug resistance associated proteins (MRPs) have been best characterized in *Arabidopsis*. Each of these MRP proteins contains at least one highly conserved ATPase domain as an energy provider (~200 aa residues long) also referred as nucleotide binding domains (NBD). This ATPase domain comprises of a Walker motif A and Walker motif B on either end of an ABC signature motif (Klein et al., 2006). MRP proteins usually contain two repeats of transmembrane (TMD) and NBD represented as TMD1-NBD1-TMD2-NBD2 (Klein et al., 2006).

Fifteen MRP transporters (*AtMRP1-15*) are reported from model plant *Arabidopsis* (Sanchez-Fernandez et al., 2001; Kolukisaoglu et al., 2002). Genome wide identification and expression analysis of ABC transporters including MRP subclass has also been explored from crop plants like rice (*OsABCC1-17*) (Jasinski et al., 2003; Garcia et al., 2004) maize (*ZmABCC1-13*) (Swarbreck et al., 2003; Pang et al., 2013) and *Vitis vinifera* (*VvABCC1-26*) (Cakier and Kilickaya, 2013). ABCC-MRP from *Arabidopsis* were shown to be involved in functions that included vacuolar sequestration of metabolites, cellular signaling (Martinoia et al., 1993), hormone transport (Ko et al., 2014), pathogen response (Ji et al., 2014; Walter et al., 2008) development of plant tissues (Wu et al., 2014), and secondary metabolite transport (Jasiñski et al., 2001). Earlier, MRP proteins were considered as the classical GSH-S conjugate pumps since evidence for other physiological roles were lacking (Ishikawa et al., 1997). Subsequently, new MRPs from *Arabidopsis* were characterized for their roles in hormonal regulation and physiological process (Gaedeke et al., 2001). Forward and reverse genetic approaches, showed their importance in the transport of glutathione and glucuronides conjugates (Liu et al., 2001), heavy metal tolerance (Gaillard et al., 2008; Park et al., 2012), chlorophyll catabolite transport (Lu et al., 1998; Tommasini et al., 1998; Frelet-Barrand et al., 2008), guard cell signaling and phytic acid transport (Nagy et al., 2009) and herbicide transport (Frelet-Barrand et al., 2008).

Studies indicated that ABCC-MRP transporters could be potential targets for trait development in higher crops (Xu et al., 2009; Panzeri et al., 2011). Embryo-specific silencing of ABC-MRP like gene resulted in the reduced phytic acid content of maize and soybean seeds (Shi et al., 2007). Recently, ABCC1 from grape berry showed their ability to transport anthocyanidin 3-*O*-glucosides *in-vitro* in an ATP- and GSH-dependent manner with high affinity (Francisco et al., 2013). A single partial wheat *ABCC-MRP* gene (AAL47686.1, partial CDS) have been speculated for xenobiotic detoxification (Theodoulou et al., 2003). Recently, another wheat MRP transporter, TaABCC3 was shown to be involved in grain development and providing resistance against secreted mycotoxin from *Fusarium* (Walter et al., 2015).

In yeast, six MRP proteins referred as Ycf1, Ybt1, Bpt1, Vmr1, Yor1, and Nft1 (Paumi et al., 2009) are present. Yeast MRPs are characterized for their possible role in the vacuolar transport of certain secondary metabolites and heavy metal sequestration (Li et al., 1996; Sharma et al., 2002). Yeast mutants defective in one or multiple *MRP* genes have been utilized as an important resource to address the functional activity of MRP orthologs across the kingdom (Tommasini et al., 1996, 1998). MRPs from plants complemented YCF1 function in yeast, thus speculating their potential involvement as a glutathione transporter (Tommasini et al., 1998; Wang and Wu, 2006).

Allohexaploid wheat (*Triticum aestivum* L.), is an important cereal crop, consumed as a staple food by a large population in the developing countries. Although, plant ABCC-MRP transporters were identified from model crops, limited attempts were made to characterize this subfamily from wheat. In this study, to address the importance of ABCC proteins for their diverse biological and physiological processes in wheat, systematic analysis and molecular function of ABCC genes was performed. Molecular gene structure and chromosomal locations of the putative wheat *ABCC* genes were predicted by both genome-wide analysis and ESTs searches. Eighteen wheat *ABCC* genes were identified and subjected to phylogenetic analyses along with *Arabidopsis* and rice counterparts. Their expression using qRT-PCR was studied to reveal their transcript accumulation in different tissues i.e., roots, stem, leaves, flag leaf, developing wheat grains, and in roots exposed to Cd stress. In addition, we demonstrated that *TaABCC13* (previously reported as *TaMRP3*, Bhati et al., 2014) has the ability to utilize adenine biosynthetic intermediates as a substrate in yeast. This is the first report providing a detailed inventory with molecular characterization of wheat ABCC-MRP transporters along with the possible functional evidence for the transport of glutathione-conjugated substrate.

## Materials and methods

### Plant materials, growth conditions, and treatments

Bread wheat cv. C306, a good processing quality Indian variety (Singh et al., 2014) was used for this study. For tissue sample collection, plants were grown in growth chambers under a 12 h photoperiod at 400 μmol m^−2^ s^−1^, 70 percent relative humidity and 25°C/18°C (day/night). Healthy plants with 14 days after anthesis (DAA) mature seeds were used to collect different vegetative tissues i.e., roots, stem, leaves, flag leaf, and seeds. Tissue samples were collected under random bulk up experiment method from different plants and snap frozen. Collected samples were stored at −80°C before RNA extraction. In total three biological replicates were used for the tissue samples. To study gene expression through seed maturation, the main individual spikes of the biological replicates were tagged at the first day after anthesis (DAA). The tagged spikes were harvested at subsequent developing days at 7, 14, 21, and 28 DAA and frozen in liquid nitrogen for RNA extraction.

For treatments, surface sterilized seeds were soaked in half strength Hoagland's solution in Petri dishes for germination. Germinating seedlings were transferred to a hydroponic growing system with half-strength Hoagland's solution. Seedlings of 7 days old (with one fully expanded leaf) were treated with Hoagland's solution supplemented with the 50 μM Cd (CdCl_2_) or 10 μM H_2_O_2_ for 24 H separately. After 24 h of stress, leaves and root samples were snapped frozen in liquid nitrogen and stored at −80°C for further use. Seedlings without stress treatment were used as the experimental control. The CdCl_2_ treated seedlings were subjected to estimate relative metal accumulation. Roots and leaves were collected and dried overnight at 60°C. The equal weight of treatment and control tissues (roots and leaves) was microwave digested in HNO_3_ (Suprapur® Merck, Germany). These digested samples were used for metal analysis by using inductively coupled plasma mass spectrometry (ICP-MS; 7700×Agilent Technologies, Santa Clara, CA).

To study the effect of hormones, abscisic acid (ABA) and gibberellic acid (GA_3_) were used as suggested earlier with minor modifications (Hwang et al., 2003; Eastmond and Jones, 2005). Six to seven spikes of 14 DAA stage were used to collect seeds and were subjected to incubation with either ABA (100 μM) or GA_3_ (60 μM) containing 20 mM CaCl_2_. Control seeds imbibed in CaCl_2_buffer were used. Three replicates containing 22–25 seeds per plates with their respective hormonal treatments were used for the experiment. After 60 min of incubation seeds were collected and rapidly chilled before storage at −80°C. RNA was extracted from the respective treatments and was subjected for qRT-PCR. Five technical replicates from biological replicates were used for qRT-PCR analysis.

### Bioinformatics analysis

To identify new ABCC-MRP subclass members from wheat, orthologous sequences from *Arabidopsis* and rice were used as query in two independent approaches. First these queries were used against the wheat EST database in tBLASTn algorithm at NCBI. The EST hits with a threshold BLAST score of 400 were considered as significant. These EST sequences were screened for presence of Walker A, B, and ABCC-MRP like ATPase domains as defined in the NCBI CDD database. The EST sequences containing typical domains were used for blast analysis on the wheat genome to map their unique position hit at different chromosome regions.

In the second approach the orthologous query sequences were individually mapped over wheat genome assemblies available at full length information for each individual member was derived from wheat genome contigs available at International wheat genome sequencing consortia (IWGSC, www.wheatgenome.org) and further these annotated ORFs were qualitatively checked with the gene structure database of wheat reported by Mayer et al. (2014). The full length amino acid sequences for each TaABCC were used to predict the domain topology and arrangement using PROSITE tool available on the ExPASy protein server. MEGA6 based alignment file prepared from all TaABCC that was used to develop an unrooted phylogenetic tree and identification of conserved amino acid patterns within TaABCC proteins. Domain prediction was performed by using Conserved Domain Database (CDD) (Marchler-Bauer et al., 2011). WebLogo3 generated logo presentations were analyzed for presence of representative ABCC-MRP domains. These identified full length sequences were named as per nomenclature guidelines and inventory of plant ABC proteins (Verrier et al., 2008). Most of these TaABCC proteins were named firstly on the basis of phylogeny and their recovery from wheat genome.

### RNA isolation and quantitative real time PCR (qRT-PCR)

RNA was extracted from different tissues like root, stem, leaf, flag leaf, and developing seeds (7, 14, 21, and 28 DAA-days after anthesis) of wheat plants. Total RNA was extracted using the RNeasy Plant MiniKit (Qiagen, Valencia, CA, USA), following manufacturer's instructions. Genomic DNA contamination was removed by DNase-I treatment (RNase free kit, Ambion, USA). Transcriptor First Strand cDNA Synthesis Kit RT-PCR (Roche, USA) was used for cDNA preparation from two micrograms of RNA. Reverse transcription was performed using random hexamer primers following the manufacturer's guidelines. Gene specific primers (Table S1) were used with QuantiTect SYBR Green RT-PCR Master mix (Qiagen, USA) based qRT-PCR reactions up to 45 cycles on ABI 7500 Fast System (Applied Biosystems, Foster City, CA, USA). Five replicates from each biological sample were used to perform qRT-PCR analysis. For PCR reaction 4–5 replicates for each gene were amplified from 3 independent cDNA preparations arising from different biological replicates. Relative expression level was quantified using 2^−ΔΔCt^ method after normalizing Ct values against 18 s rRNA expression (Schmitteng, 2001). The data are expressed as mean ± standard deviation. Statistical analysis was performed using Origin 6.0 software (Origin Lab Corporation, MA, USA). One-Way analysis of variance (ANOVA) followed by Turkey's multiple comparison test was applied to check the level of significance. In all the tests, statistical significance was checked at *p* < 0.01 and/or *p* < 0.05.

### Phenotypic complementation of yeast adenine biosynthetic mutants and quantitation of pigmentation

Yeast strain, YPH499 (ABC154) defective in adenine biosynthesis pathway, with a genotype of *MATα, ura3–52; leu2*- Δ *1; lys2–801; his3* Δ *200; trp1* Δ *63; ade2–101* was used as a positive control. Yeast mutant for *ycf1* (ABC 470) with genotype of *MATα, Dycf1::KanMX2; ura3–52; leu2-D1; lys2–801; his3D200; trp1D63; ade2–101* prepared in the same auxotrophic background was also used for the current study (37). For complementation experiment, *TaABCC13 +* pYES263 harboring in yeast Δ*ycf1* mutant was used. Pre-culture of wild type, mutant and transformed yeast colonies were grown overnight in YPD broth and brought to the same OD_600_. The colonies were subsequently streaked on 0.5% YPD media for 4 days along with negative controls. The red pigmentation produced by the yeast colonies that includes *ade2* mutants was quantified spectrophotometrically as described earlier (Chaudhuri et al., 1997). Briefly, yeast cells were grown to the saturation in half strength YPD to an OD of 2.0. Equal amount of cells were further lysed using glass beads in 5% sulfosalicyclic acid. The amount of pigmentation was measured at the absorbance of 530 nm.

## Results

### Inventory, molecular structure, and phylogenetic analysis of wheat ABCC proteins

EST databases and genome assembly of wheat were used to identify candidate ABCC-MRP genes. In total ~75 wheat ESTs was screened from dbEST BLAST results. ESTs were assembled to identify possible partial or full ORF with conserved domains and were designated as new TaABCC members. The result of BLAST analysis identified AAL47686.1 and AAQ10074.1 as previously annotated partial ABCC proteins from wheat. In addition to that, TaMRP3 (AIK23242.1) was also identified previously and characterized in wheat tissues (Bhati et al., 2014). Subsequently, the information generated from the recently released wheat genome was also utilized to identify more wheat ABCC-MRPs (Mayer et al., 2014). Our refined searches resulted in identification of 18 ABCC subclass proteins from wheat, hereafter referred as TaABCC1–TaABCC18. The nomenclature was based on the guidelines those are widely accepted for plant ABC protein (Verrier et al., 2008). Following this, previously reported *TaMRP3* gene (Bhati et al., 2014) has now been referred as *TaABCC13*.

**Figure 1:**
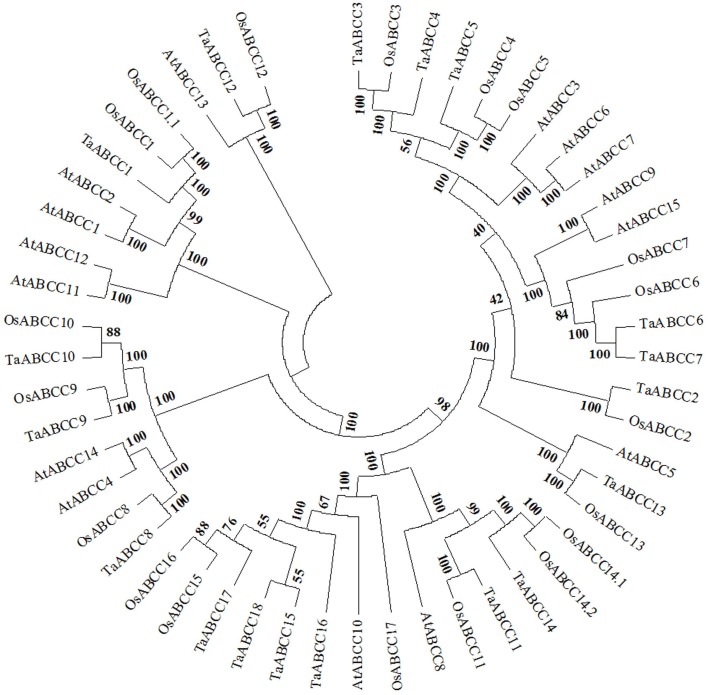
**Phylogeny alignment of plant ABCC-MRP proteins**. Phylogenetic tree analysis of different MRP genes identified from wheat (TaABCC1-18), *Arabidopsis* (AtABCC1-15), rice (OsABCC1-17) (Updated sequence accessions with the new systematic names were used from Verrier et al., 2008).

To assign the possible functional clues to wheat ABCC proteins, the identified wheat ABCC protein sequences were subjected to phylogenetic analysis along with ABCC-MRP members from *Arabidopsis* and rice. Based on our analysis each of the cluster consisted of multiple ABCC from *Arabidopsis* and rice suggesting a wide distribution of wheat ABCC proteins (Figure 1). Among wheat ABCC, TaABCC15 showed highest homology with TaABCC17 with percentage identity of 88.2. On the contrary, highest divergence was observed between TaABCC2 and *TaABCC3* with percentage identity of 29.8. When a cross species comparison was done, the highest percentage identity of 75.1 was observed for TaABCC5 and AtMRP5. Similarly with rice, the highest percentage identity of 87.6 was observed for TaABCC6 with OsMRP6. Interestingly, TaABCC3 and TaABCC4 belong to the group that included a Cd inducible AtMRP3 (Bovet et al., 2003). Low phytic acid homolog genes TaABCC13 (previously reported as TaMRP3, Bhati et al., 2014) and AtMRP5 are grouped together along with OsMRP13 (Figure 1).

**Figure 2:**
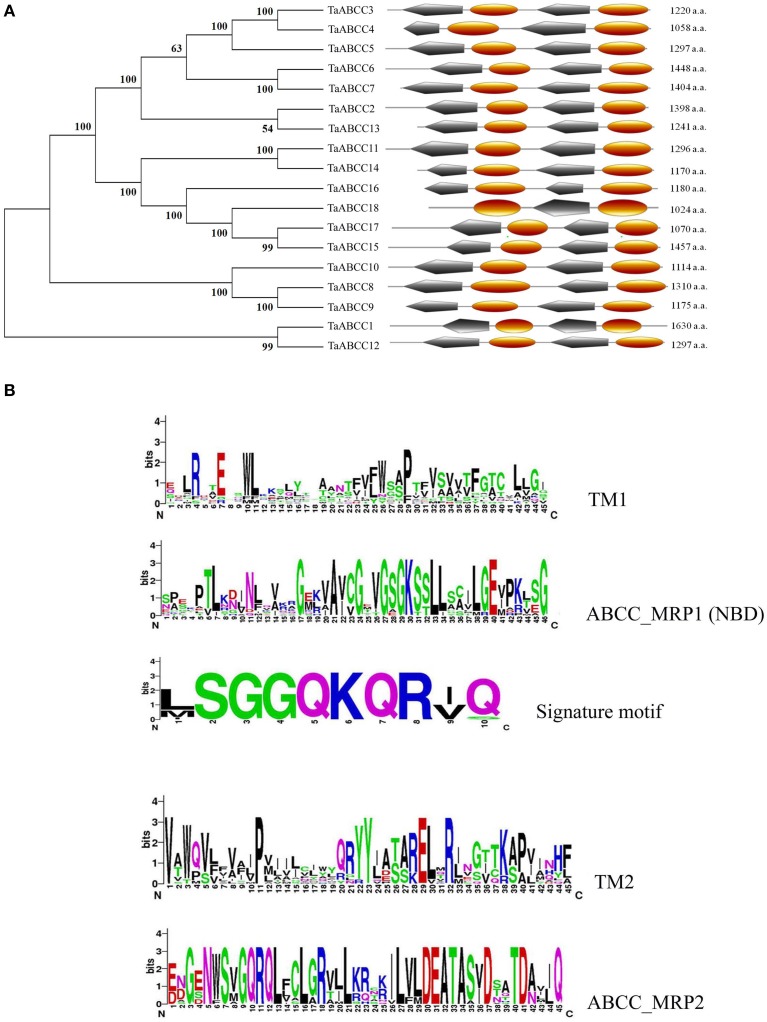
**Phylogenetic tree for TaABCC proteins based on the neighbor joining method, schematic diagram of the domain arrangement in different TaABCC and logo representation of amino acid conserved in different domains. (A)** The un-rooted phylogenetic tree (left side) from TaABCC amino acid sequences was developed using the NJ method on MEGA6 software and domain representations (right side) were prepared using PROSITE server, TM- Transmembrane domain, MRP- nucleotide binding domain from Multidrug Resistant associated Protein). **(B)** Multiple alignment was analyzed on WebLogo 3 server from University of California, Berkeley for logo representation of TM, ABCC_MRP domains and signature motif; the Y-axis represents conservation of amino acid at that position (height).

Translated amino acid sequences for each TaABCC confirms presence of Signature motif and functional domains like transmembrane (TM) and ABCC-MRP (NBD) like domain those are conserved ubiquitously among members of ABCC-MRP transporter subclass. The predicted full length amino acid sequences from all TaABCC proteins were analyzed on CDD and Expasy PROSITE. The analysis revealed specific signatures of TM-NBD-TM-NBD with the possible location on membrane except for TaABCC members, without any mapped EST (Figure 2A). A ClustalW based sequence alignment was used to create logo representation for signature motif and each conserved domain. Logo representation strongly suggests more conserved amino acid in ABCC-MRP domains as compared to transmembrane domains (Figure 2B). ABCC-MRP1 domain in all wheat ABCC proteins has conserved NBD represented by K-S/T-S/T amino acids. ABC signature motif (SGGQKQR) was conserved and present among all the wheat ABCC proteins in the NBD domain of ABCC-MRP transporters (Figure 2B).

### Genomic distribution of ABCC genes on wheat genomes

Genomic evolution of modern wheat is contributed by three different diploid parents i.e., *T. urartu, Ae. speltoides, and Ae. tauschii* (contributed A, B, and D genome, respectively) (Mayer et al., 2014). The isolated ORFs for *TaABCC* genes were used to map their representative chromosomal locations based on contig assembly at IWGSC BLAST server (http://wheat-urgi.versailles.inra.fr). The CDS sequences for selective wheat *ABCC* genes were further confirmed by 5′ or 3′ RACE sequencing and subsequently refined by using IWGSC for their chromosomal locations (Table 1). Each *TaABCC* genes was mapped on all three homoeologous wheat chromosome but with varying similarity score. Genomic coordinates for each wheat *ABCC* genes and their homoeologous are mentioned in Table S2. All the wheat *ABCC* genes were also analyzed for their genomic structure using IWGSC sequences. Result suggested the presence of multiple introns (ranging from 2 to 27) for wheat ABCC genes (Figure S1).

**Table 1:** **Inventory and chromosomal location of ABCC genes from wheat**.

**Gene**	**Representative ESTs**	**Chromosomal Location**
TaABCC1^*^	HX108911.1, DR735499.1, BJ224413.1, CJ805925	2AL, 2BL, 2DL
TaABCC2	CJ691783.1, CJ585444.1	3AL, 3BL, 3DL
TaABCC3^*^	CJ699639.1, HX193542.1, CV762434.1	3AS, 3DS, 4AS
TaABCC4^*^	CJ956417.1, CD877047.1, CJ944377.1,	3AL, 3BL, 3DL
TaABCC5	None Found with significant similarity score	1AL, 1BL, 1DL
TaABCC6^*^	CJ859948.1, HX191927.1, CJ668043.1, CJ856118.1,	2AL, 2BL, 2DL
TaABCC7	None Found with significant similarity score	2AL, 2BL, 2DL
TaABCC8^*^	CJ668035.1, HX159331.1, AL822018.1, BQ620567.1	5BL, 5DL, 5AL
TaABCC9	BJ309016.1, CJ649956.1, CJ541775.1	2AS, 2BS, 2DS
TaABCC10	None found with significant similarity score	2DS, 2AS, 2BS
TaABCC11^*^	CD884377.1, HX070437.1, CD865647.1, CJ690695.1	7AL, 7BL, 7DL
TaABCC12	HX143310.1	7AS, 7BS, 7DS
TaABCC13^*^	CD910572.1, CD910572.1, CA730883.1, HX147655.1	5AL, 4BL, 4DL
TaABCC14	CJ667633.1, CJ560395.1, BJ303226.1	3AS, 3BS, 3DS
TaABCC15^*^	HX159319.1, CJ656810.1, CJ689124.1	7AS, 7BS, 7DS,
TaABCC16^*^	CV760609.1, CJ656810.1, HX159319.1, CJ689124.1	7AS, 7BS, 7DS,
TaABCC17	CJ667633.1, CJ560395.1, BJ303226.1	3A, 3B, 3D
TaABCC18	None found with significant similarity score	7AS, 7BS, 7DS

* *Indicates the ABCC genes for which sequence information was confirmed by either 5′ or 3′ RACE. Underline indicates the primers amplifying the transcript arising from these genomes*.

### Differential expression patterns of ABCC genes in wheat

**Figure 3:**
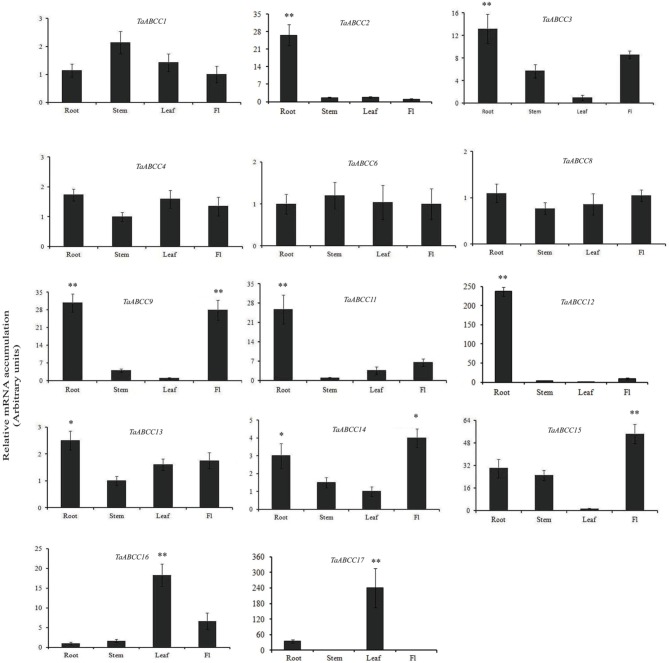
**Expression analysis of wheat ABCC genes in different parts of wheat plants**. qRT-PCR analysis was performed on the cDNA templates prepared from 2 μg of DNase free RNA isolated from roots (R), stem (S), leaves (L), and flag leaf (FL) of 14 DAA wheat plants. Relative transcript accumulation of the genes was calculated. Each bar indicates the mean of five replicates with the indicated standard deviation of the mean. ^**^ indicates significant difference at *p* < 0.01. ^*^ indicates significant difference at *p* < 0.05.

In order to gain insight into the transcript accumulation of wheat *ABCC* transporter in different tissues, qRT-PCR analysis was performed. Expression studies were planned for those genes which are either covered by multiple reliable EST or present in our in-house transcriptome assembly from multiple wheat tissues. No unique representative ESTs (dbEST) was observed for *TaABCC5*, *TaABCC7, TaABCC10*, and *TaABCC18*; hence they were not selected for the expression study. The expression pattern of the remaining wheat *ABCC* genes was studied in roots, stem, leaves, and flag leaf that could help in speculating their site of molecular function. Analysis of the transcript abundance revealed that all the genes displayed differential expression patterns across the set of wheat tissue samples. Expression data, suggested the induction of wheat *ABCC* genes in leaves, root, stem, and flag leaf at different folds (Figure 3).

The transcript levels for *TaABCC8* and *TaABCC6* showed no significant change in expression levels and are ubiquitously expressed in all the tissue studied. Remaining *TaABCC* genes showed differential expression responses. Interestingly, expression of *TaABCC4* and *TaABCC13* were observed in similar patterns for all the tissues, although at different folds. In roots transcript accumulation was observed highest for *TaABCC12* (~220-fold) followed by *TaABCC2*, *TaABCC11* (~25-fold), and *TaABCC3* (~12-fold) (Figure 3). *TaABCC9* and *TaABCC14* showed predominant expression in both roots and flag leaf. *TaABCC16* and *TaABCC17* are expressed at significantly higher level in leaves compared other tissues (Figure 3). Overall, expression data suggested differential and overlapping expression patterns implicating their function in wheat.

### Temporal expression of wheat ABCC genes in seeds

**Figure 4:**
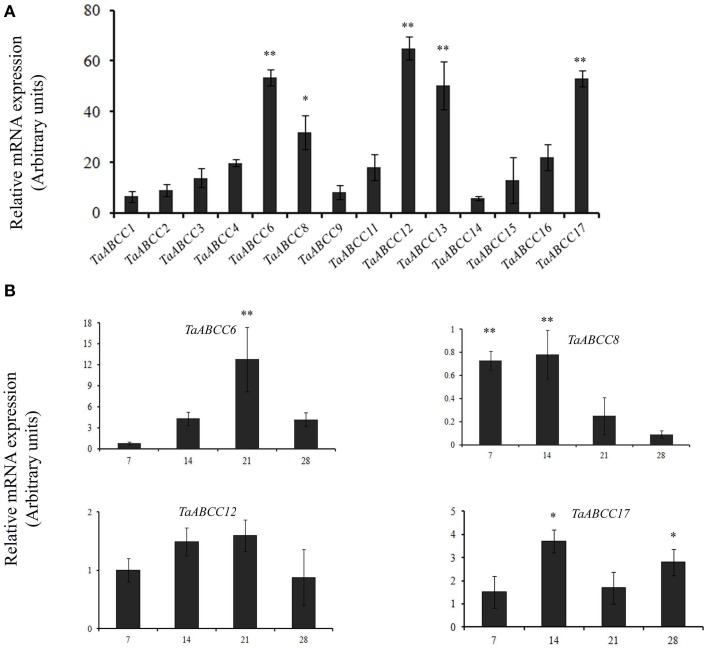
**Quantitative expression of wheat ABCC genes in wheat seeds. (A)** The cDNA templates were prepared from 2 μg of DNase free RNA isolated from wheat seeds of 14 DAA. Each bar indicates the mean of 4–5 replicates with the indicated standard deviation of the mean. **(B)** qRT-PCR analysis during wheat seed development. The cDNA templates were prepared from 2 μg of DNAse free RNA isolated from different time point of seed maturation at 7, 14, 21, and 28 DAA. Each bar indicates the mean of five replicates with the indicated standard deviation of the mean. ^**^ indicates significant difference at *p* < 0.01. ^*^ indicates significant difference at *p* < 0.05.

In order to characterize *TaABCC* genes in seeds, quantitative expression analyses were performed in 14 DAA wheat seeds. This time point represents a complete differentiation of outer aleurone layer and endosperm tissue of wheat seed. Expression data suggested significantly higher expression for *TaABCC6, TaABCC8, TaABCC12, TaABCC13*, and *TaABCC17* in wheat seeds (Figure 4A). The relative transcript accumulation of these transcripts ranges ~30− to ~65-fold. Among the expressed wheat *ABCC* genes, relative transcript abundance of *TaABCC12* was highest in seed (Figure 4A).

Temporal expression analysis of highly expressed *ABCC* genes in wheat was performed at different stages of grain filling (i.e., 7, 14, 21, and 28 DAA). *TaABCC13* analysis was excluded since it was previously reported (previously reported as *TaMRP3*, Bhati et al., 2014). Only *TaABCC8* was highly expressed at the early time points of grain filling compared to other wheat *ABCC* genes (Figure 4B). The expression of *TaABCC8* decreased further with the maturation of the wheat seeds. *TaABCC6* was significantly induced at 21 DAA compared to that of other time points. Overall, expression analysis suggested that all the *TaABCC* genes responds differentially during the development of wheat seed.

### Hormonal regulation of ABCC genes

**Figure 5:**
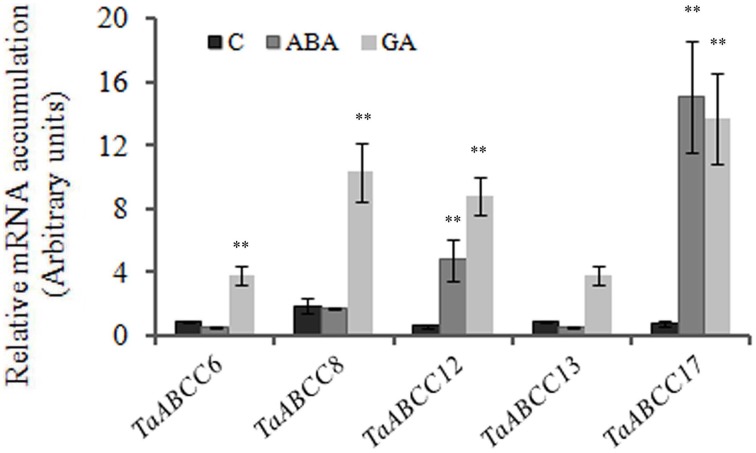
**Hormonal regulation of wheat ABCC genes**. Fourteen DAA old wheat seeds were treated with either ABA (100 μM) or GA_3_ (60 μM) for 60 min. Seeds were collected and were subjected for RNA isolation. For quantification, cDNA templates were prepared from 2 μg of DNase free RNA isolated from wheat tissue. Each bar indicates the mean of five replicates with the indicated standard deviation of the mean. ^**^ indicates significant difference at *p* < 0.01 with respect to control.

The selected *ABCC* genes those are highly expressed during wheat seed development were further studied for their response to exogenous treatment of GA_3_ and abscisic acid (ABA). In the current study, we chose GA_3_ and ABA, since these are the two major hormones that control the dynamics of seed maturation and grain filling (Sreenivasulu et al., 2006; Thiel et al., 2008). Expression analysis of genes in 14 DAA seeds when exposed to ABA showed no significant changes in the transcript accumulation of *TaABCC8* (Figure 5A). The transcript abundance of *TaABCC6* and *TaABCC13* was slightly repressed. Only *TaABCC12* and *TaABCC17* were induced by ABA and GA_3_. In contrast, upon GA_3_ treatment, transcript accumulation of *TaABCC6*, *TaABCC8*, *TaABCC12 TaABCC13*, and *TaABCC17* was significantly increased with respect to the control (Figure 5B). These results suggested that wheat *ABCC* genes are preferentially induced in seeds; when exogenously treated with GA_3_.

### Expression patterns of ABCC genes under cadmium stress

**Figure 6:**
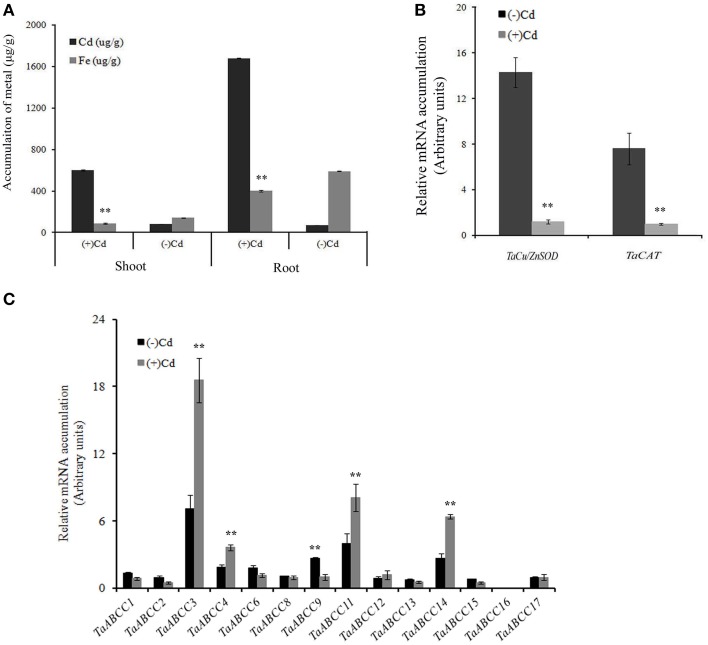
**Effect CdCl_2_ on expression of wheat** ***ABCC*** **genes. (A)** Cd accumulation in shoots and roots of 3 weeks old wheat seedlings when exposed to CdCl_2_. **(B)** qRT-PCR analysis of *TaCu/ZnSOD* and *TaCAT* when exposed to roots of wheat seedlings. **(C)** qRT-PCR analysis of all the identified wheat *ABCC* genes in roots of wheat seedlings exposed to CdCl_2_. For quantification, cDNA templates were prepared from 2 μg of DNAse free RNA isolated from wheat roots. Each bar indicates the mean of five replicates with the indicated standard deviation of the mean. ^**^ indicates significant difference at *p* < 0.01 with respect their control.

Heavy metal (HM) toxicity is one of the major abiotic stresses leading to hazardous effects in plants. Multiple plant ABCC-MRPs were speculated for their functional role for vacuolar sequestration, thus reducing the cellular metal toxicity (Bovet et al., 2003; Gaillard et al., 2008). In order to characterize the *TaABCC* against the metal stress, we exposed the wheat seedlings to Cd. After exposure, Cd accumulation was measured in roots and shoots of wheat seedlings. Results showed significantly higher accumulation of Cd in the plants exposed to metal compared to their respective controls (Figure 6A). In roots, Cd accumulation was 2.5-fold higher when compared to shoots (Figure 6A). Previously, it has been suggested that the plants exposed to Cd are a poor accumulator of iron (Meda et al., 2007). During our study, we also measured the iron accumulation in roots and shoots of Cd treated wheat seedlings. Our result suggested a significant decrease in the uptake of iron in roots treated with Cd (Figure 6A). No significant changes in the accumulation of iron in shoots were observed, suggesting the possibility that in this study most of the Cd gets retained in the roots rather being transported in the shoots within the time frame of the experiment.

The highest concentration of Cd exposure to the plants is usually accompanied with the interference in the expression of *TaCu/ZnSOD* and catalase transcripts (*TaCAT*) (Qiu et al., 2013). Thus, quantitative real time PCR (qRT-PCR) expression analysis of *TaCu/ZnSOD* transcripts was checked. Down-regulation of *TaCu/ZnSOD* and *TaCAT* transcript was observed in presence of Cd suggesting its toxic effect (Figure 6B). To study molecular responses to Cd, wheat *ABCC* genes were studied for their relative expression analysis in roots. Based on the expression of *TaABCC* genes, they could be classified into two categories; those which are differentially regulated and others where no significant change in the transcript accumulation was observed. The relative transcript levels among wheat *ABCC* genes suggested an enhanced expression of four genes i.e., *TaABCC3*, *TaABCC4*, *TaABCC11*, and *TaABCC14* when exposed to Cd (Figure 6C). The highest transcript accumulation was observed for *TaABCC3* that showed an increased transcript abundance of two- to three-folds when compared to control roots. Surprisingly, some of the wheat *ABCC* genes showed suppression in the expression. This suppression was observed consistently among the biological replicates. These slightly suppressed genes included *TaABCC1*, *TaABCC6*, and *TaABCC9*. No significant changes in the expression levels were observed for *TaABCC2, TaABCC8, TaABCC12, TaABCC13, TaABCC16*, and *TaABCC17* (Figure 6C).

### Expression patterns of ABCC genes in presence of H_2_O_2_

**Figure 7:**
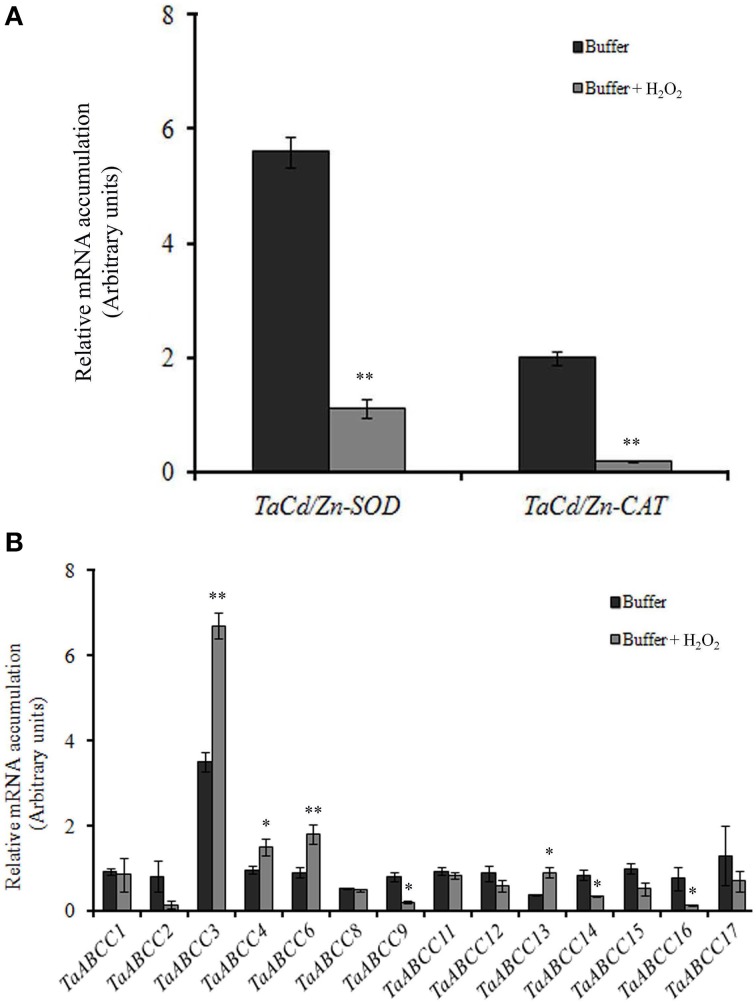
**Effect of H_2_O_2_ on expression of wheat** ***ABCC*** **genes. (A)** qRT-PCR analysis of *TaCu/ZnSOD* and *TaCAT* when the roots of wheat seedlings were exposed to H_2_O_2_. **(B)** qRT-PCR analysis of all the identified wheat *ABCC* genes in wheat seedlings exposed to H_2_O_2_. The cDNA templates were prepared from 2 μg of DNA-free RNA isolated from the roots of the wheat seedlings. Each bar indicates the mean of 4–5 replicates with the indicated standard deviation of the mean. ^**^ indicates significant difference at *p* < 0.01 with respect to control. ^*^ indicates significant difference at *p* < 0.05 with respect to control.

Previous reports suggested that exposure of plants to abiotic stress such as Cd causes accumulation of H_2_O_2_ (Cho and Seo, 2005). The induction of *TaABCC* transcripts when exposed to Cd could be also possibly due to generation of oxidative stress. To address this, wheat seedlings were exposed to oxidative stress (H_2_O_2_). Results showed down-regulation of *TaCu/ZnSOD* and *TaCAT* transcript when wheat seedlings were exposed to H_2_O_2_ (Figure 7A). Expression data suggested that few of the *TaABCC* genes were highly up-regulated in presence of H_2_O_2_. qRT-PCR analysis indicated an increased transcript accumulation of *TaABCC3*, *TaABCC4*, *TaABCC6*, and *TaABCC13* in roots when exposed to H_2_O_2_ (Figure 7B). Upon H_2_O_2_treatment, transcript accumulation was highest for *TaABCC3* with the cumulative abundance of two-fold higher when compared to control roots. On the contrary, no significant changes in the transcript mRNA accumulation were observed for *TaABCC1*, *TaABCC8*, *TaABCC11, TaABCC12*, and *TaABCC17*. Interestingly, transcript expression of *TaABCC2, TaABCC9, TaABCC14*, and *TaABCC16* were slightly reduced (Figure 7B). These results suggested that Cd induced accumulation of H_2_O_2_ might be responsible for enhanced transcript abundance of certain wheat *ABCC* genes.

### TaABCC13 is involved in transport of glutathione-conjugates in yeast

**Figure 8:**
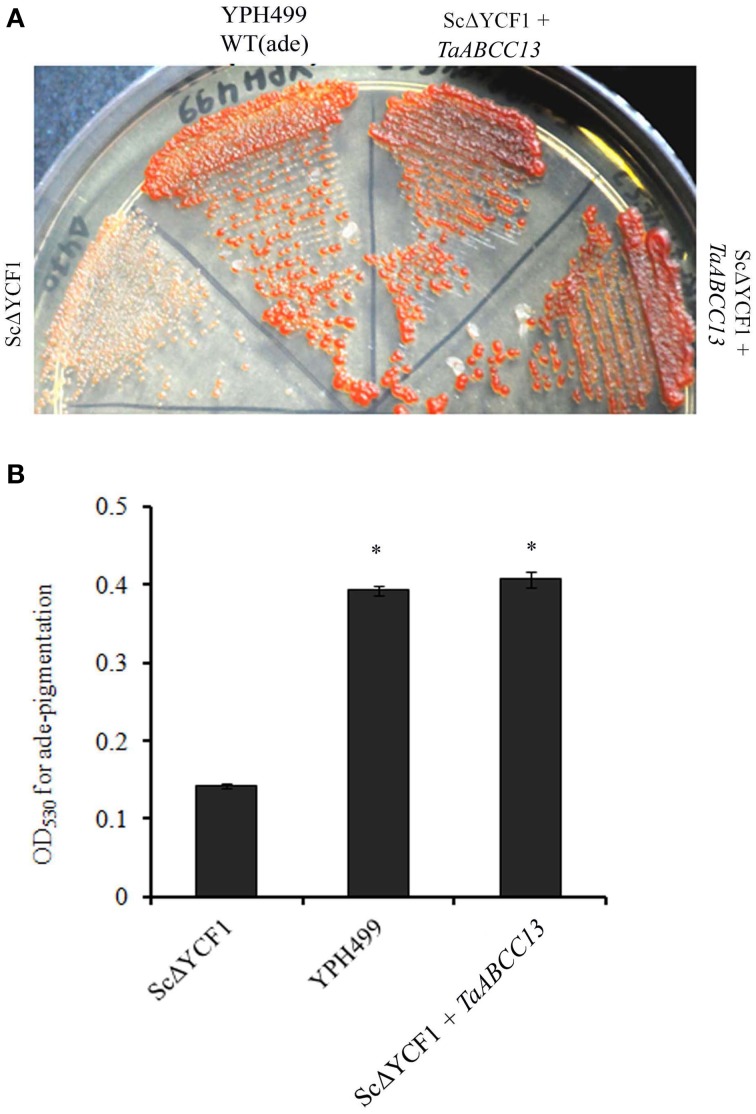
**Phenotypic complementation of yeast YCF1 mutant with TaABCC13 under adenine-limiting condition. (A)** Parent strain YPH299 and its *Sc*Δ*ycf1* were used to complement with TaABCC13. All the cultures were used to complement with TaABCC13. All the cultures were grown to the OD of 0.5 and subsequently streaked on YPD plates with 0.5% of yeast extract. For control, *Sc*Δ*ycf1* transformed with empty plasmid pYES263 was used. All the strain used was isogeneic for nutritional marker. Pictures were taken 4 days post-incubation at 30°C. **(B)** Quantification of red pigmentation for mutant and *Sc*Δ*YCF1* complemented with TaABCC13 was performed on the cell culture grown under adenine limiting conditions as described earlier. Each bar indicates the mean of four replicates with the indicated standard deviation of the mean. ^*^ indicates significant difference at *p* < 0.01 with respect to control.

Previously, *TaABCC13* (earlier*TaMRP3*, Bhati et al., 2014) was shown to complement yeast Δ*ycf1* mutant for its Cd sensitivity. Yeast MRP proteins, Ycf1p and Bpt1p (Li et al., 1996; Petrovic et al., 2000) are the two members that are known to be involved in transport of glutathione-conjugates and have different degree of sensitivity toward Cd (Klein et al., 2000; Sharma et al., 2002). Based on these studies we suspected that TaABCC13 should also has the ability to detoxify glutathione-conjugates. To ascertain the role in glutathione-mediated detoxification by MRP, phenotypic assays for pigment accumulation in adenine biosynthetic mutants has been utilized (Sharma et al., 2003). Yeast Δ*ycf1* when complemented with TaABCC13 could rescue the red pigmentation under adenine limiting conditions (Figure 8A). In order to calculate the extent of complementation, assays were performed to measure the intensity of pigmentation in yeast (Sharma et al., 2003). Our data suggested a significant accumulation (~2.5-folds) of the pigmentation in the wild type and yeast Δ*ycf1* complemented with TaABCC13 compared to control (Figure 8B). These results confirm the ability of TaABCC13 to transport toxic adenine biosynthetic intermediates (phosphoribosyl-amino-imidazole and phosphoribosyl-amino-imidazole carboxylate) in Δ*ycf1* those are conjugated with glutathione.

## Discussion

In the past, there have been significant efforts to understand the multiple functions of ABC transporters in yeast, humans and recently in plants. Although their roles in model plants have been explored extensively, but such evidences are still largely obscure from crops like wheat. Recent reports from rice, maize, and grapes conferred valuable roles to ABC transporters and offers initiative for further characterizing their biological implications. With advancement in understanding the functions of ABCC-MRP subclass, it became evident that they act as major detoxifiers and sequester metal-chelators into the plant vacuoles (Martinoia et al., 1993). Role of wheat ABCC-MRP proteins were not largely addressed till date. This work led to the identification of 18 wheat MRPs that were characterized in detail for their gene expression.

### Gene structure and genomic distribution of wheat ABCC

ABCC-MRP subclass represents one of the important groups of ABC transporters that contains N-terminal extension TMD domain. This study identified 18 full length ABCC proteins. The largest identified protein was TaABCC1 with 1630 aa whereas the smallest one TaABCC18 contains 1024 aa (Table 1). Similar size distribution was also observed for rice and *Arabidopsis* (Verrier et al., 2008). Wheat being a three genome system, it was anticipated to have three homoeologous of representative genes (Table 1). All identified genes were located on either of the chromosome1, 2, 3, 4, 5, and 7 (Table 1). We failed to recover the full length sequence for few homoeologous due to limited wheat genome contig information. Nonetheless, considering the presence of homoeologous in wheat, this sums to a total of 54 wheat *ABCC* genes. The recent draft of wheat genome has also annotated putative members of ABCC-MRP subfamily (Mayer et al., 2014). Thus, analysis of the draft annotation sequences for ABCC-MRP like transporters was done and summarized along with their sequence identifier (Table S3). Interestingly, performing in-depth analysis of the above data resulted in the multiple annotations for the same protein sequences of varying lengths. Alternatively, utilizing EST and transcriptome based strategies to identify the expressed genes can be one of the reliable approaches for a complex and challenging genome like wheat. The IWGSC genome analysis suggested maximal wheat ABCC annotated sequences mapped to chromosome set 7, chromosome set 2, and chromosome set 3 of wheat genome. Four of the wheat *ABCC* genes are located on the chromosome 7 along with their respective homoeologous, showing high density region for wheat ABCC. This represents the comparatively high density of ABC-MRP annotation on chromosome 7, which can be as a result of intra-chromosomal duplication events during evolution of hexaploid wheat genome (Mayer et al., 2014). Our analysis of chromosomal location matches with the IWGSC annotation report. Contrarily, the current study was based on manual gene annotation using the ESTs and a genome contig database which takes into account the expression of the genes and addresses their functional roles in wheat. Furthermore, upon careful examination, no ESTs were mapped from NCBI for *TaABCC5*, *TaABCC7*, *TaABCC10*, and *TaABCC18*. Based on our previous observation, it seems some of these wheat *ABCC* genes may not be expressed, although careful experiments are required to confirm the expression of these transcripts.

In a few cases, this work reflects a more comprehensive observation compared to that of IWGSC annotations and gene structure predictions. During our analysis varying lengths of ABCC proteins were ascertained, but truncated *TaABCC18* was identified as pseudogene. Such annotated truncated or pseudo genes were previously reported in case of *Arabidopsis* (Sanchez-Fernandez et al., 2001) and maize (Pang et al., 2013).

### Wheat ABCC genes showed differential expression patterns

Studying the expression patterns in specific tissues and organs suggests the molecular clues for their role and help to address their functionality in plants. The preferential expression patterns suggested their specificities for the respective tissue/organs. For example, *TaABCC2* and *TaABCC3* showed highest expression in the roots whereas *TaABCC1* and *TaABCC16* are preferentially expressed in the stem and leaves. Phylogenetic analysis suggested that AtABCC6 and TaABCC3 are present in the same cluster. *AtMRP6* is highly expressed at the initiation point of secondary roots, especially in xylem-opposite peri-cycle cells where lateral roots initiate (Gaillard et al., 2008). Growing roots are active sites for the synthesis of auxins (Ljung et al., 2005). *TaABCC3* is also a close match for *AtMRP3* and both the genes were found to be induced by HMs (Brunetti et al., 2015). Further *TaABCC3* was induced by the presence of GA_3_, indicating its role in root architecture development, although further functional studies are required for confirmation. Further, examining the promoter of *TaABCC3* derived from Chromosome 3B, resulted in identification of regions those are associated with root specific expression (GSH transporter and DRE), GA_3_ (GA-Myb) regulated domains and phosphate responsive domain (P1BS) (data not shown). This suggests that *TaABCC3* can be a prime candidate for plant genetic engineering to enhance tolerance against heavy-metal accumulation.

*TaABCC14* and *TaABCC15* are highly abundant in flag leaf and both are clustered together in the phylogenetic tree. Flag leaf is the important organ for the biosynthesis of transitory starches, micronutrients and other metabolites that contribute to the seed maturation during the senescence (Ali et al., 2010). These observations suggested an important role of specific ABC transporters in flag leaf. TaABCC1 forms a distinct cluster with OsABCC1 and showed high expression in stem, suggesting its preferred site of function. *OsABCC1* was recently shown to be involved in arsenic tolerance by sequestering it into the node cell vacuoles (Song et al., 2014). Both *TaABCC1* and *OsABCC1* are not induced by the presence of Cd. Similar expression profiles and close clustering on the phylogeny tree might indicate *TaABCC1* may also perform such roles in plant tissue.

The early expression of these genes during seed development suggested their possible roles for seed trait development. Seed maturation is a result of controlled flux of hormones (GA_3_, ABA, and ethylene) and nutrients between the seed tissues (Sreenivasulu et al., 2006; Thiel et al., 2008). Thus, possible impact of the GA_3_ and ABA was tested on the transcript accumulation of wheat ABCC genes that are highly expressed in seeds. Earlier a PDR1-like gene was shown to be repressed by ABA treatment (Zhang et al., 2013) likewise in our case *TaABCC6* and *TaABCC13* were also repressed (Figure 5). Most of the wheat ABCC genes were highly induced by GA_3_, which is supported by previous expression pattern observed for *AtABCC13*/*ABCC11* for this hormone (Guizani et al., 2014). The differential expression response of wheat ABCC genes for hormone treatment will help in assimilating clues regarding their possible roles in integrated pathways that are involved in multiple abiotic stresses. TaABCC13 is the closest functional ortholog for the OsMRP13 and AtMRP5 those are involved in phytic acid transport. TaABCC13 is a functional protein that could be also possibly involved in PA transport, especially in the aleurone tissue of the seed (Bhati et al., 2014). Thus, targeting *TaABCC13* by recently developed genome editing tools will be a viable strategy to generate low phytate crop in wheat. On the similar grounds, the functionality of some of the other wheat *ABCC* genes could be assessed by using yeast or *Arabidopsis* mutants.

In this study, response of wheat *ABCC* genes for Cd mediated response was also addressed. During our experiments, Cd accumulation was found in roots and aerial parts of wheat plants, thus transcriptional regulation of genes involved in Cd transport and detoxification is expected. Down regulation of *Cu/ZnSOD* transcripts upon Cd stress is in accordance with the response observed previously in wheat, and *Arabidopsis* (Cuypers et al., 2011; Qiu et al., 2013). On the contrary, induction of *Cu/ZnSOD* transcripts was observed in soybean and perennial rye-grass suggesting that regulation of antioxidant activities those are induced by Cd is dependent on the plant species (Clemens, 2001). Uptake of other micronutrient especially Fe gets perturbed when plant roots were exposed to HMs like Cd (Astolfi et al., 2014). Similarly, in our study, uptake of iron was decreased in the presence of Cd (Figure 6A). The reduced support for the uptake of Fe and other micronutrient is explained due to “inducible deficiency” or because of Cd induced regulation of Fe-homeostasis (Cuypers et al., 2011). The expression of wheat *ABCC* genes was observed when exposed to either Cd or H_2_O_2_. Based on these results, it is suggested that the regulation of wheat ABCC could be either due to the direct effect of Cd or through the H_2_O_2_ generated by toxicity. Interestingly, *TaABCC11* was only specifically induced by Cd and no significant change in expression was observed when exposed to H_2_O_2_. For the remaining wheat *ABCC* genes it seems that H_2_O_2_ generated by the Cd toxicity might be responsible for differential gene expression.

### Conservation of functional activity of wheat ABCC

Yeast defective in ycf1 (yeast cadmium factor) an MRP transporter has been classically utilized for confirming the functional role of ABCC-MRP transporters from plants (Tommasini et al., 1998; Wang and Wu, 2006; Bhati et al., 2014). Earlier we reported that, *TaABCC13* (earlier referred as TaMRP3) rescued Δ*ycf1* sensitivity toward cadmium but its functional activity in utilizing glutathione-conjugates were not studied (Bhati et al., 2014). Under adenine limiting conditions, the toxic intermediates phosphoribosyl-amino-imidazole and phosphoribosyl-amino-imidazole carboxylate gets accumulated in these WT (*ade1/ade2*: YPH499) mutant. These toxic metabolites are conjugated with glutathione and transported inside the vacuole primarily by YCF1 (Sharma et al., 2003) showing red pigmentation. Therefore, defective YCF1 showed significant decrease in phenotypic pigmentation. Based on the pigmentation assays, the current study confirms the ability of TaABCC13 in transport of toxic adenine biosynthetic intermediates by the accumulation of red-pigmentation in YCF1 yeast mutant (Figure 8). Based on the PSort analysis, TaABCC13 reliability score for membrane localization was similar to that of ScYCF1, ZmLpa1, AtMRP1, AtMRP2, AtMRP5 and PvMRP1 (data not shown). AtMRP1 and AtMRP2 complements yeast YCF1 mutants as well as actively influx glutathione-conjugated substrate into the plant vacuoles (Martinoia et al., 1993; Liu et al., 2001). Similarly, ABCC1 from grape berry is also able to transport glutathione dependent substrates (Francisco et al., 2013). Based on these observations, it is evident that TaABCC13 could detoxify Cd and is also involved in the membrane transport of glutathione-conjugated substrates by sequestration. Thus, TaABCC13 is involved in a conserved function that a classical ABCC-MRP are known to perform. Surprisingly, no significant change in the expression of *TaABCC13* was observed when exposed to Cd at 24 h. Thus, it could be important to study the expression of *TaABCC13* when exposed to different doses of Cd. In roots, *TaABCC3* was highly up-regulated when exposed to Cd toxicity. It would be valuable to screen remaining wheat *ABCC* genes for the functional rescue of Δ*ycf1* sensitivity for Cd.

The current study provided the spatial-temporal characterization of 18 wheat *ABCC* genes. Wheat ABCC genes showed differential expression in the presence of Cd or H_2_O_2_ suggesting their importance in abiotic stresses. Additionally, TaABCC13 was able to rescue the functional activity of yeast mutant by utilizing glutathione-conjugated substrates. The insight provided herein will be a much needed foundation regarding the wheat ABCC protein function that could be directed for cereal crop improvement.

### Conflict of interest statement

The authors declare that the research was conducted in the absence of any commercial or financial relationships that could be construed as a potential conflict of interest.

## Supplementary material

Figure S1 **Exon/intron structures of wheat ABCC genes are shown**. Black boxes represent exons and black lines represent introns. Total number of introns for each gene are represented on the right side.

## Abbreviations

ABC-MRP: ATP binding cassette multi drug resistance protein
DAA: day after anthesis
PA: phytic acid
lpa: low phytic acid.
