# Supplementary figures and images for: Genome-wide identification and expression characterization of ABCC-MRP transporters in hexaploid wheat
Source: Front Plant Sci. 2015 Jul 1;6:488. doi: 10.3389/fpls.2015.00488 (PMC4486771; doi:10.3389/fpls.2015.00488)

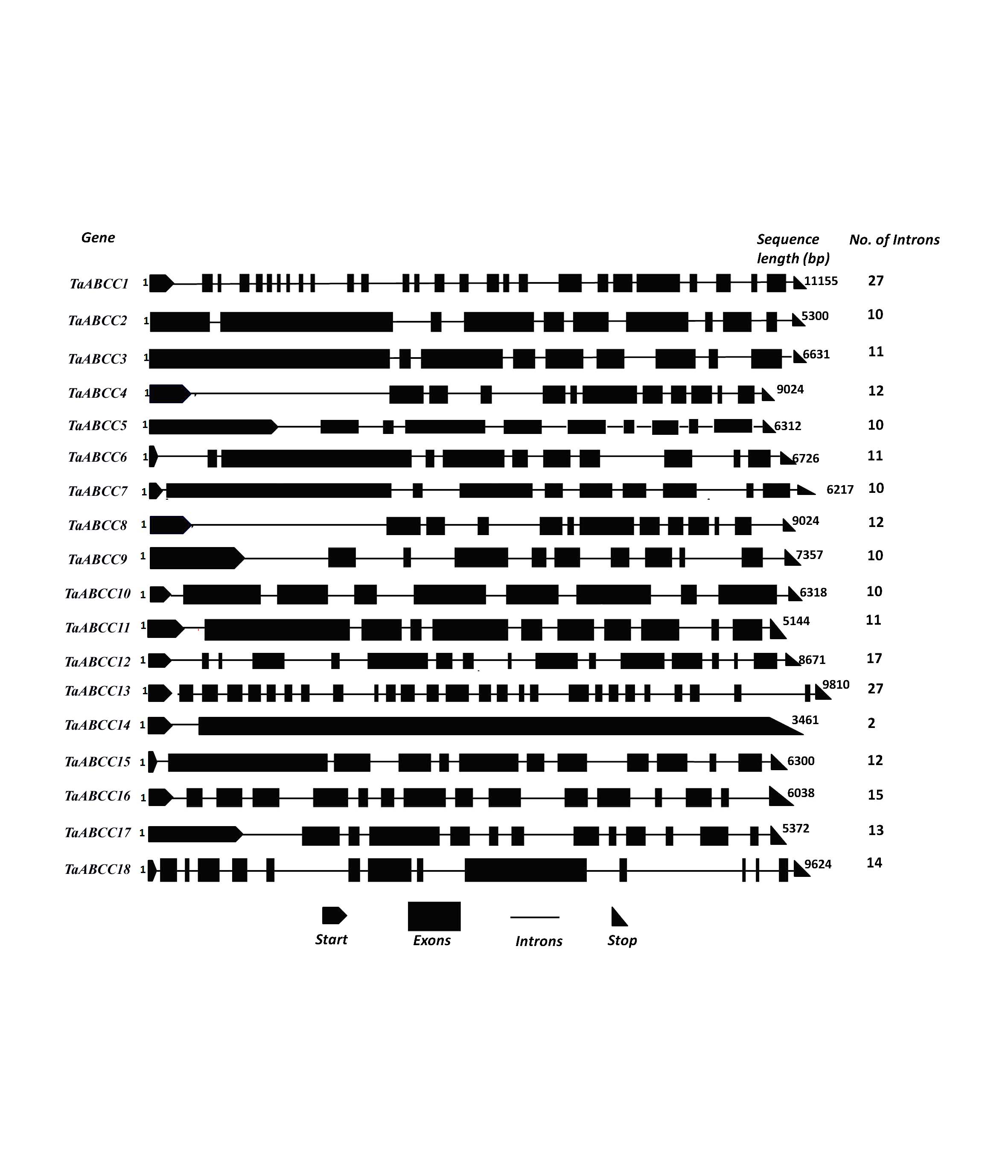

Supplement: Figure S1 — Exon/intron structures of wheat ABCC genes are shown. Black boxes represent exons and black lines represent introns. Total number of introns for each gene are represented on the right side. [file Image1.JPEG]
