# Supplementary material for: Genome-wide identification and expression characterization of ABCC-MRP transporters in hexaploid wheat
Source: Front Plant Sci. 2015 Jul 1;6:488. doi: 10.3389/fpls.2015.00488 (PMC4486771; doi:10.3389/fpls.2015.00488)
Supplement: Supplementary file 2 [file Table1.DOCX]

**Table S1|** List of primers used in the study

| **Name of Gene** | **Primers**  **(5’-3’)** | **Amplicon size**  **(in bp)** |
| --- | --- | --- |
| *TaABCC1* | Forward: GTCTGTCCACAATCCGTGCTT  Reverse: TCATGATGCCACCCAATGTT | 150 |
| *TaABCC2* | Forward: GGGAGTGCATGATTTGCGGTGTAGG  Reverse: TA CCC TGG CCA AGC ACA CAA GTT GCC | 224 |
| *TaABCC3* | Forward: TTGCTAGCAATTACTGGATGGCGTG  Reverse: TGGAATCGAAGAAAGACATAGAGC | 224 |
| *TaABCC4* | Forward: CATTGCTAGCAATTACTGGATGGC  Reverse: GATGCGCCCACTTGGAGTGGAATC | 250 |
| *TaABCC6* | Forward: TCCGAGCTCCCATGTCCTT  Reverse: CCCCAATGGTTCCCAGAATT | 150 |
| *TaABCC8* | Forward: GACAGATGGAATTCATGTAAACAG  Reverse: TCTGGGATATTTGCCCAGCTAAGA | 203 |
| *TaABCC9* | Forward: AGGTCTGGTGAAGGAATTCGATGC  Reverse: GAGGTTGGACGAGCGGTC | 103 |
| *TaABCC11* | Forward: CATTCTGTGTCGGGGCATGATAGA  Reverse : TGCATTTTGAGCTAGTTCTCTCCATC | 279 |
| *TaABCC12* | Forward: ATTGTCCATCTTCATCACAGTTGAAATGCGG  Reverse: CTTGATGAGTGCACAGCCAATGTTGACAATCA | 132 |
| *TaABCC13* | Forward: ATGGCTCTGCCTGCGAATGGA  Reverse: CAAGGCCAGCCATACTTGGT | 110 |
| *TaABCC14* | Forward: ACCACTCTCCTGAGCGCG  Reverse: AGAGGATCGACGTTGCTCCTTAC | 173 |
| *TaABCC15* | Forward: GACAGATGGAATTCATGTAAACAG  Reverse: TCTGGGATATTTGCCCAGCTAAGA | 203 |
| *TaABCC16* | Forward: CATTCTGTGTCGGGGCATGATAGA  Reverse : TGCATTTTGAGCTAGTTCTCTCCATC | 279 |
| *TaABCC17* | Forward: GTG GAA CGG GTG AAC CAG TAC ATG G  Reverse: AGCATCTTTCCTATACCTGATCTTCAAATCTC | 129 |
| *TaCuZnSod* | Forward: TTGTTGGGAGAGCGTTTGTTGTTC  Reverse: AAAACCAGAGATGGAAACCAGCGA | 132 |
| *TaCat* | Forward: CAACAACCACTACGACGGGCTCAT  Reverse: CTGTTGATGAATCGCTCTTGCCTT | 230 |
| *Ta18SrRNA* | Forward: GTGACGGGTGACGGAGAATT  Reverse: GACACTAATGCGCCCGGTAT | 150 |
