# Supplementary material for: Genome-wide identification and expression characterization of ABCC-MRP transporters in hexaploid wheat
Source: Front Plant Sci. 2015 Jul 1;6:488. doi: 10.3389/fpls.2015.00488 (PMC4486771; doi:10.3389/fpls.2015.00488)
Supplement: Supplementary file 3 [file Table2.DOCX]

**Table S2|** Genomic information and contigs for each TaABCC genes. *TaABCC* genes were mapped on wheat genome draft sequences using BLASTn algorithm. Each of the *TaABCC* was matched at multiple chromosomal locations, confirming the presence of homeolog sequences.

| **Gene** | **Wheat genome sequence survey Contig Identifier** |
| --- | --- |
| *TaABCC1* | >lcl\|5375621_17573_1055084_2AL_ab_k71  >lcl\|7975309_6234_189658_2270982_6027986_2BL_ab_k71  >lcl\|9875944_23839_873258_6228975_4189086_2DL_ab_k71 |
| *TaABCC2* | >lcl\|4449444_7121_91345_4249227_4234181_3AL_ab_k71  >lcl\|10356358_19419_602520_3329137_8460072_3B_ab_k71  >lcl\|6932093_5692_43444_4764498_6827562_6295507_3DL_ab_k71 |
| *TaABCC3* | >lcl\|3370051_6254_60914_1422993_2012582_3AS_ab_k71  >lcl\|5951722_7034_577989_1867548_1377307_4AS_v2_ab_k71  >lcl\|7639166_6698_160603_3B_ab_k71 |
| *TaABCC4* | >lcl\|4435621_12554_102507_3950209_2204050_3AL_ab_k71  >lcl\|10443277_11132_294121_1423959_9193533_3BL_ab_k71  >lcl\|6831742_4851_39248_1651790_3132514_3DL_ab_k71 |
| *TaABCC5* | >lcl\|3931622_4780_197342_2257675_3138549_1AL_v2_ab_k71  >lcl\|3796108_15180_1620967_175154_2381501_1BL_ab_k71  >lcl\|2256316_10450_198841_1114971_667679_1DL_ab_k95 |
| *TaABCC6* | >lcl\|6399751_12037_476241_4485535_6296680_2AL_ab_k71  >lcl\|8006379_8778_277882_3869060_2201781_2BL_ab_k71  >lcl\|9821209_10917_224147_26096_9715111_2DL_ab_k71 |
| *TaABCC7* | >lcl\|6435883_12135_288262_6296680_683632_2AL_ab_k71  >lcl\|8006380_20777_862822_3869060_2064389_2BL_ab_k71  >lcl\|9910117_8326_114881_9804938_26096_2DL_ab_k71 |
| *TaABCC8* | >lcl\|2696750_1279_5526_1123198_471266_1618456_5AL_ab_k95 >lcl\|10765929_12964_169875_8530575_8984082_5BL_ab_k71  >lcl\|4498102_5514_69015_415971_3593987_5DL_ab_k71 |
| *TaABCC9* | >lcl\|5189613_18155_920555_308096_868174_534259_2AS_ab_k71  >lcl\|4748675_8185_154999_2BS_ab_k71  >lcl\|5377037_7948_485647_4464866_272439_2DS_ab_k71 |
| *TaABCC10* | >lcl\|5154109_14007_427716_571542_4000086_2AS_ab_k71  >lcl\|5232804_25722_674754_4606302_1507892_2BS_ab_k71  >lcl\|5368504_10320_355422_3834487_3441925_2DS_ab_k71 |
| *TaABCC11* | >lcl\|4389618_938_3026_1665222_4357449_7AL_ab_k71  >lcl\|6724187_8716_75179_5678001_2715270_7BL_ab_k71 >lcl\|3393581_19739_499599_3294100_3062574_7DL_ab_k71 |
| *TaABCC12* | >lcl\|4253855_17893_219200_4122210_4078885_7AS_ab_k71 >lcl\|3129856_17094_346317_2021882_2810607_7BS_ab_k71 >lcl\|3966335_21600_459492_3826164_3808143_7DS_ab_k71 |
| *TaABCC13* | >lcl\|2668947_5630_68742_10969_288133_5AL_ab_k95  >lcl\|7041386_11700_94169_6935361_2292025_4BL_ab_k71  >lcl\|4DL_14452637_4DL_v3_ab_k71 |
| *TaABCC14* | >lcl\|3332170_2952_23779_432244_3307891_3292451_3AS_ab_k71  >lcl\|10749463_4366_26940_10138040_10367616_3BS_ab_k71  >lcl\|2585749_692_2376_1620923_1788484_3DS_ab_k95 |
| *TaABCC15* | >lcl\|4252760_15601_205408_4112688_2707888_7AS_ab_k71  >lcl\|3081718_3930_123771_542792_498917_7BS_ab_k71  >lcl\|3898642_5888_125133_1731307_1286562_7DS_ab_k71 |
| *TaABCC16* | >lcl\|4252760_15601_205408_4112688_2707888_7AS_ab_k71  >lcl\|3097754_17569_365534_1032872_196845_7BS_ab_k71  >lcl\|3898642_5888_125133_1731307_1286562_7DS_ab_k71 |
| *TaABCC17* | >lcl\|4249017_14991_143955_4081004_3766419_7AS_ab_k71  >lcl\|4209728_14022_166671_2133978_3866299_7BS_ab_k71 >lcl\|3897159_9117_260684_1685064_3536716_7DS_ab_k71 |
| *TaABCC18* | >lcl\|4252760_15601_205408_4112688_2707888_7AS_ab_k71  >lcl\|3097754_17569_365534_1032872_196845_7BS_ab_k71  >lcl\|3869315_10011_201622_787661_3808710_7DS_ab_k71 |
