# Supplementary material for: Genome-wide identification and expression characterization of ABCC-MRP transporters in hexaploid wheat
Source: Front Plant Sci. 2015 Jul 1;6:488. doi: 10.3389/fpls.2015.00488 (PMC4486771; doi:10.3389/fpls.2015.00488)
Supplement: Supplementary file 4 [file Table3.DOCX]

**Table S3|** Compilation of **s**equence identifier for the wheat ABCC genes and their respective amino acid sequence. Survey sequence, gene models for TaABCC transporters were retrieved from the genome annotation (Mayer et al., 2014) and was checked against our manual TaABCC annotations. Amino acid sequences listed are represented by their unique sequence identifier.

| **Gene** | **>Sequence identifier followed by amino acid sequence**  **As reported by IWGSC (Mayer et al., 2014)** |
| --- | --- |
| *TaABCC1* | >Traes_2BL_2E1918E7F.1  MARAVYSDSDVYLFDDPLSALDAHVGRQVFDKCIKEELRHKTRVLVTNQLHFLPYVDKILLIHDGVVKEEGTFDELSNTGEQFKKLMENAGKMEEQTEEKQDENKSQDDIKHTENGDVVIADGGPQKSQDSSSKTKQGKSVLIKQEERETGVVSTKVLSRYKNAMGGIWAVSVLFLCYTLTETLRISSSTWLSIWTDEGSLNIHGPGYYNLIYGILSFGQVLVTLTNSYWLITSSLRAAKRFSKDLGDIDRNLAVFVNMFMAQISQMLSTFVLIGVVSTMSLWAIMPLLILFYAAYLYYQATSREVKRMDSITRSPVYAQFSEALNGLSTIRAYKAYDRMSNINGKSMDNNIRFTLVNMSSNRWLAIRLETLGGIMIWFTATFAVMQNQRAENQKAFASTMGLLLTYTLNITNLLTAVLRLASLAENSMNAVERVGTYIELPSEAPPVIEDNRPPPGWPSSGIIKFEDVVLRYRPELPPVLHGISFIINGSEKVGIVGRTGAGKSSMLNALFRIVELERGRILIDDCDTSKFGIWDLRKVLGIIPQAPVLFSGTIRFNLDPFSEHNDADLWEALERAHLKDVIRRNALGLDAEVSEAGENFSVGQRQLLSLARALLRRAKILVLDEATAAVDVRTDALIQKTIREEFKSCTMLIIAHRLNTVIDCDRLLILSSGKISEFDTPENLLSNEDGAFSKMVQSTGPSNAEYLKSLVLGNGEERLRKEESKMQDIQRKWAASNRWAVAAQFALAASLASSHSDLLSLEVAEGNNILRKTKDAVLTLQGVLEGKHNTEIEESLTEYQVPSDRWWSSLYKVIEGLATMSKLGRNRLRQPGYSFENHGSIDWDQI  > Traes_2AL_0A38A0EFC.1  MGFEPLEWYCQPVKDGAWSRAMESAFGAYTPCGIDSLVVCISYLALFGVCFYRIWRTTKDYKVQRYKIRSPYYNYLLGLLVVYCIAEPLYKIATGTSIMNLDGQSGLAPFEVTSLVIEIAAWCCMLTMILLETKIYITEFRWYIRFVVIYVLVGKAAMFNVVLPVRQYYSSSSIFYLYCSEIICQCVFGILMVVYLPSLDPYPGYTPIRSELLVDDNTDYEPLPGGEQICPERHANIFSRIFFSWMTPLMQQGYKRPITDSDIWKLDDWDETETLYNRFQECWNKELQKPKPWLLRALHSSLGGRFWLGGFFKIGNDASQFVGPTVLSLLLESMQKGDPSWNGYIYAFSIFAGVSLGVLAEAQYFQNVMRTGFRLRSTLIAAVFRKSLRLTNDSRKKFASGRITNLISTDAESLQQVCQQLHSLWSAPFRIVIAMVLLYAQLGPAALLGALMLALLIPIQTVIIGKMQKLTKEGLQRTDKRISLMNEILAAMDTVKCYAWEQSFQSKVQDIRDDELSWFRSAQLLAALNSFILNSIPVVVTVVSFGVYSLLGGELTAAKAFTSLSLFAVLRFPLFMLPNLITQVVNCKVSLKRLEDLLLADERILMPNPPIDPELPAISIKNGNFSWELQAERPTLSNVNLDVPVGSLVAIVGSTGEGKTSLISAMLGEIAPVSGSDTSVVIRGSVAYVPQVSWIFNATVRDNILFGSPFQPSRYGRAIDSTALRHDLDLLPGGDLTEIGERGVNISGGQKQRVSMARAVYSDSDVYLFDDPLSALDAHVGRQVFDKCIKEELRHKTRVLVTNQLHFLPYVDKILLIHDGVVKEEGTFDELSNTGEQFKKLMENAGKMEEQTEEKQDENKSQDDIKHTENGDVVIADGGLQKSQDSSSKTKQGKSVLIKQEERETGVVSTKVLSRYKNAMGGIWAVSVLFLCYTLTETLRISSSTWLSIWTDEGSLNIHGPGYYNLIYGILSFGQVLVTLTNSYWLITSSLRAAKRLHDYMLRSILRAPMVFFHTNPLGRIINRFSKDLGDIDRNLAVFVNMFMAQISQLLSTFVLIGVVSTMSLWAIMPLLILFYAAYLYYQATSREVKRMDSITRSPVYAQFSEALNGLSTIRAYKAYDRMSNINGKSMDNNIRFTLVNMSSNRWLAIRLETLGGIMIWFTATFAVMQNQRAENQKAFASTMGLLLTYTLNITNLLTAVLRLASLAENSMNAVERVGTYIELPSEAPPVIEDNRPPPGWPSSGIIKFEDVVLRYRPELPPVLHGISFIINGSEKVGIVGRTGAGKSSMLNALFRIVELERGRILIDDCDTSKFGIWDLRKVLGIIPQAPVLFSGTIRFNLDPFSEHNDADLWEALERAHLKDVIRRNALGLDAEVSEAGENFSVGQRQLLSLARALLRRAKILVLDEATAAVDVRTDALIQKTIREEFKSCTMLIIAHRLNTVIDCDRLLILSSGKISEFDTPENLLSNEDGAFSKMVQSTGPSNAEYLKSLVLGNGEERLRKEESKLQDIQRKWAASNRWAVAAQFALAASLASSHSDLLSLEVAEGNNILRKTKDAVITLQGVLEGKHNTEIEESLTEYQVPSDRWWSSLYKVIEGLATMSKLGRNRLRQPGYSFENHGSIDWDQI  > Traes_2DL_5F40C7E14.1  MGFEPLEWYCQPVKDGAWSRAMESAFGAYTPCGIDTLVVCISYLALFGVCFYRIWRTTKDYKVQRYKIRSPYYNYLLGLLVVYCIAEPLYKIATGTSIMNLDGQSGLAPFEVTSLVIEIAAWCCMLTMILLETKIYITEFRWYIRFVVIYVLVGKAAMFNVVLPVRQYYSSSSIFYLYCSEIICQCVFGILMVVYLPSLDPYPGYTPIRSELLDDNTDYEPLPGGEQICPERHANIFSRIFFSWMTPLMQQGYKRPITDSDIWKLDDWDETETLYNRFQECWNKELQKPKPWLLRALHSSLGGRFWLGGFFKIGNDASQFVGPTVLSLLLESMQKGDPSWNGYIYAFSIFAGVSLGVLAEAQYFQNVMRTGFRLRSTLIAAVFRKSLRLTNDSRKKFASGRITNLISTDAESLQQVCQQLHSLWSAPFRIVIAMVLLYAQLGPAALLGALMLALLIPIQTVIIGKMQKLTKEGLQRTDKRISLMNEILAAMDTVKCYAWEQSFQSKVQDIRDDELSWFRSAQLLAALNSFILNSIPVVVTVVSFGVYSLLGGELTAAKAFTSLSLFAVLRFPLFMLPNLITQVVNCKVSLKRLEDLLLADERILMPNPPIDPELPAISIKNGNFSWELQAERPTLSNVNLDVPVGSLVAIVGSTGEGKTSLISAMLGEIAPVSGSDTSVVIRGSVAYVPQVSWIFNATVRDNILFGSPFQPSRYGRAIDSTALRHDLDLLPGGDLTEIGERGVNISGGQKQRVSMARAVYSDSDVYLFDDPLSALDAHVGRQVFDKCIKEELRHKTRVLVTNQLHFLPYVDKILLIHDGVVKEEGTFDELSNTGEQFKKLMENAGKMEEQTEEKQDENKSQDDIKHTENGDVVIADGGLQKSQDSSSKTKQGKSVLIKQEERETGVVSTKVLSRYKNAMGGIWAVSVLFLCYTLTETLRISSSTWLSIWTDEGSLNIHGPGYYNLIYGILSFGQVLVTLTNSYWLITSSLRAAKRLHDYMLRSILRAPMVFFHTNPLGRIINRFSKDLGDIDRNLAVFVNMFMAQISQLLSTFVLIGVVSTMSLWAIMPLLILFYAAYLYYQATSREVKRMDSITRSPVYAQFSEALNGLSTIRAYKAYDRMSNINGKSMDNNIRFTLVNMSSNRWLAIRLETLGGIMIWFTATFAVMQNQRAENQKAFASTMGLLLTYTLNITNLLTAVLRLASLAENSMNAVERVGTYIELPSEAPPVIEDNRPPPGWPSSGIIKFEDVVLRYRPELPPVLHGISFIINGSEKVGIVGRTGAGKSSMLNALFRIVELERGRILIDDCDTSKFGIWDLRKVLGIIPQAPVLFSGTIRFNLDPFSEHNDADLWEALERAHLKDVIRRNALGLDAEVSEAGENFSVGQRQLLSLARALLRRAKILVLDEATAAVDVRTDALIQKTIREEFKSCTMLIIAHRLNTVIDCDRLLILSSGKISEFDTPENLLSNEDGAFSKMVQSTGPSNAEYLKSLVLGNGEERLRKEESKLQDIQRKWAASNRWAVAAQFALAASLASSHSDLLSLEVAEGNNILRKTKDAVITLQGVLEGKHNTEIEESLTEYQVPSDRWWSSLYKVIEGLATMSKLGRNRLRQPGYSFENHGSIDWDQI |
| *TaABCC2* | > Traes_3AL_D19BE3FD1.1  MKIMEAKDSRIKAMAEAMRSMRILKLHAWETAYLDKLLKLRDVEKGWLRKYLYTCSAIAFLFWASPTLVSVVTFGVCILVEIPLSAGTVLSALATFRILQDPIYNLPELVSMVTQTKVSLDRIEEFIKEDHQGKPSCHGNVNGTKDLAMAGEIVIEPGEYSWEADTSSKKTKVTLKINSKVSIGKGLKVAVCGPVGSGKSSLLYSIMGEIPRVSGAEPMVVGSRAYVPQSAWIQTGTIQDNVLFGKAMDRSLYEEVLQGCALDRDLEIWANGDMTVVGERGVNLSGGQKQRIQLARALYSDSDVYFLDDPFSAVDAHTSAHLFKECLLRLMSSKTVMYVTHQLEFLRDSDLVLVMKDGRIVQSGKYDDLIADKDGELSKQMAAHDQSLSQVNPAKAHGLPKSKKQKKQIEATEIESDGHVIGRECEEERESGRVKWDVYRKFVTSAYGGGLIPVVLLCQVFFQGLQICSNYWIAWAAEREDQASTDQATVDTDIPYRLAGLIFAMIQLLSIIFIMSQIAWPIFMLFIIIIAISTWYQNYYISSARELARMVGIRKAPVLHHFSETVTGAATIRCFNQGEEFLTKSLALIDDYTRITFHNSATVEWLCIRINFLFNLVFFVMLIILVSLPRDTIDPSLAGLAATYGLNLNVLQAWVIWNLCNVENKMISVERIFQFSNIPSESPLVIENSRPRETWPWCGTIQIEALQIQYSPDMPMVLKGISCTFPGERKIGVVGRTGSGKSTLIQALFRVVEPSAGRIFIDEVDISLLGVHDLRCRLSIIPQEPTLFQGTVRTNLDPLQQHLDPEIWEVLHKCCLEEIVREDNRLLDAPVVEDGGNWSVGQRQLVCLARVLLMKKKILVLDEATASVDTATDNIIQKTIRQETDNCTVITIAHRIPTVIDSDLVLVLGEGRILEFDSPENLLRDESSAFSKLVMEFVGRSEGRHQ  > Traes_3B_064551019.1  MKIMEAKDSRIKAMAEAMKSMRILKLHAWETAYLDKLLKLRDVERGWLRKYLYTCSAIAFLFWASPTLVSVVTFGVCILVEIPLSAGTVLSALATFRILQDPIYNLPELVSMVTQTKVSLDRIEEFIKEDHQGKPSCHGNINGMKDLAMAGEIIIEPGEYSWEADTSSKKTKVTLKINSKVSIRKGLKVAVCGPVGSGKSSLLYSIMGEIPRVSGAEPTVVGSRAYVPQSAWIQTGTIQDNVLFGKAMDRSLYEEVLQGCALDRDLELWANGDMTVVGERGVNLSGGQKQRIQLARALYSDSDVYFLDDPFSAVDAHTSAHLFKECLLRLMSSKTVMYVTHQLEFLRDSDLVLVMKDGRIVQSGKYDDLIADKDGELSKQMAAHDQSLSQVNPAKAHGLPKSKKQKKQIEATEIESDGHVIGRECEEERESGRVKWDVYRKFVTSAYGGGLIPVVLLCQVLFQGLQICSNYWIAWAAEREDQASTDQATIDTDIPYRLAGLIFAMIQLLSIIFIMSQIAWPIFMLFIIIIAISTWYQSYYISSARELARMVGIRKAPVLHHFSETVSGAATIRCFNQGEKFLTKSLALIDDYTRITFHNSATVEWLCIRINFLFNLVFFVMLVILVSLPRDTIDPSLAGLAATYGLNLNVLQAWVIWNLCNVENKMISVERIFQFSNIPSESPLVIENSRPRETWPWCGTIQIEALQIQYSPDMPMVLKGISCTFPGERKIGVVGRTGSGKSTLIQALFRVVEPSAGRIFIDGVDISLLGVHDLRCRLSIIPQEPTLFQGTVRTNLDPLQQHLDTEIWEVLRKCRLEEIVREDNRLLDAPVVEDGGNWSVGQRQLVCLARVLLMKKKILVLDEATASVDTATDNIIQKTIRQETDNCTVITIAHRIPTVIDSDLVLVLEEGRILEFDSPENLLRDESSAFSKLVMEFVGRSEGRH |
| *TaABCC3* | > Traes_3B_9A61DFBAA.1  MGPLLAVGNKKALGLDDVPDLDHADSVAGLLPSFKTNLEAQAGDGSGPKFTAFKLTKALVRTVWWHIAVTALYALIYNLATYVGPYLIDSLVQYLNGDERYASKGKLLVVTFIVAKVFECLSQRHWFFRLQQAGIRARSALVSVVYQKGLSLSSISRQSRTSGEMINIISVDADRVGLFSWYMHDLWLVPLQVGMALFILYSTLGVASLAALGATIVVMLANVPPMKMQEKFQQKLMDCKDVRMKATSEILRNMRILKLQGWEMKFLSKIIDLRTTETSWLKKYLYTSTAATFVFWGAPTFVAVVTFGACMLLGIPLESGKVLSALATFRVLQEPIYNLPDTISMMIQTKVSLDRIASFLCLEELPTDAVERLPSGSSNVAIEVSNGCFSWDGSPELPTLKDLNFEAQQGMRVAVCGTVGSGKSSLLSCILGEVPKLSGEVKTCGTMAYVSQTAWIQSGKIQDNILFGKEMDSEKYDRVLEWCSLKKDLEILPFGDKTVIGERGINLSGGQKQRIQIARALYQDADIYLFDDPFSAVDAHTGSHLFKECLLGALASKTVVYVTHHIEFLPSADLILVMKGGRIAQAGKYNDILGSGEELMELVGAHQDALTALDVIDVANGGSETISSSLSRSLSSAEEKDKQSGKDNGDKVQSGQLVQEEEREKGRVGFWVYWKYLTLAYGGALVPFVLIAQLLFQVLQIASNYWMAWASPVSKDAEPPVSTSTLIYVFVALAVASSLCILIRALFLVTAAYKTATLLFNKMHMAIFRAPMSFFDSTPSGRILNRASTDQSEVDTNIAYQMGSVAFSIIQLVGIIAVMSQVAWQVFLVFVPVIIICFWYQRYYIETARELQRLVGVCKAPIIQHFAESITGSTTIRSFGKEHQFVSTNSHLMDAYSRPKFYNAAAMEWLCFRLDTLSSFTFAFALVFLISLPTGIIDPGIAGLAVTYGLNLNMLQAWVVWSMCNLENKIISVERILQYISIPEEPPLSMSEDKLPHNWPSQGEIQLRDVHVRYAPQLPFVLKGLNVTFPGGMKTGIVGRTGSGKSTLIQALFRIVEPTVGQILVDGVDICTIGLHDLRSRLSIIPQDPTMFEGTVRSNLDPLNEYNDDQIWEALDNCQLGDEVRKKELKLDSPVIENGENWSVGQRQLVCLGRVILKRTKILVLDEATASVDTATDNMIQKTLRENFSEATVITIAHRITSVLDSDMVLLLDNGVAVERDTPAKLLENKSSLFSKLVAEYTIRATHA  > Traes_3DS_EE5D85DE2.1  MKGGRIAQAGKYNDILGSGEELMELVGAHQDALTALDVIDVANGGSETISLSLSRSLSSAEEKDKQNGKDDGDKVQSGQLVQEEEREKGRVGFWVYWKYLTLAYGGALVPFVLIAQLLFQVLQIASNYWMAWASPVSKDAEPPVSTSTLIYVFVALAVASSLCILIRALFLVTAAYKTATLLFNKMHMAIFRAPMSFFDSTPSGRILNRASTDQSEVDTNIAYQMGSVAFSIIQLVGIIAVMSQVAWQVFLVFVPVIIICFWYQRYYIETARELQRLVGVCKAPIIQHFAESITGSTTIRSFGKEHQFVSTNSHLMDAYSRPKFYNAVAMEWLCFRLDTLSSFTFAFALVFLISLPTGIIDPGIAGLAVTYGLNLNMLQAWVVWSMCNLENKIISVERILQYISIPEEPPLSTSEDKLPSNWPSEGEIQLRDVHVRYAPQLPFVLKGLNVTFPGGMKTGIVGRTGSGKSTLIQALFRIVEPTVGQILVDGVDICTIGLHDLRSRLSIIPQDPTMFEGTVRSNLDPLNEYNDNQIWEALDNCQLEDEVRKKELKLDSPVIENGENWSVGQRQLVCLGRVILKRTKILVLDEATASVDTATDNMIQKTLRENFSEATVITIAHRITSVLDSDMVLLLDNGVAVERDTPAKLLENKSSLFSKLVAEYTMRATHT |
| *TaABCC4* | > Traes_3DL_BE884863B.1  MDSEKYDRVLELCSLKKDLESFPSGDQTVIGERGINLSGGQKQRVQIARALYQDADIYLFDDPFSAVDAHTGSHIFKECLLGALAQKTVLYVTHQLEFLPAADLILVIKDGVIAQSGRYNDILSSGEEFMQLVGAHQDALAAIDAIDVPNGASEAFSSSDAASLSGSLPSADKKDKQNVKQDDGHGQSGQLVQEEERERGRVGFWVYWKYLTLAYGGALVPFVLLAQMLFEVLHIASNYWMAWAAPASKDVEPPVSMYTLIYVYVALALGSSVCTFVRALFLVPAAYKTATLLFNKMHVSIFRAPMSFFDSTPSGRILNRASTDQSLVDTSIANRMGSIAFAFIQLGGTIVVMSQVAWQVFVVFIPVIAICLWYQRYYIDTARELQRMVGICKAPIIQHFVESITGSTIIRSFGKENQFLSTNNQLMDAYSRPKFYNAGAMEWLCFRMDMLSSLTFAISLIFLINLPTGIIDPGIAGLVVTYGLNLNIMQVTLVTSMCNLENKIISVERILQYLSLPEEAPLSMSEDGLAHNWPSEGEIQLHNLHVKYAPQLPFVLKGLTVTFPGGMKTGIVGRTGSGKSTLIQALFRIMDPTIGQITVDGVDICTIGLHDLRSRLSIIPQDPTMFDGTVRHNLDPLGEYTDNQIWEALDHCQLGDEVRRKELKLDSPVVENGENWSVGQRQLVCLGRVILRRTKILVLDEATASVDTATDNLIQKTLQQHFSGATVITIAHRITSVLHSDIVLLLDNGMAVEHQTPARLLEDKSSLFSKLVAEYTMRSTRT  > Traes_3B_292E8BD00.1  MITFIFWSAPTFIAVVTFGACVLMGIPLESGKVLSALATLRVLQESIYNLPDRISAIIQTKVSLDRIASFLCLEEFPTDAVQKLPIGSSDVAIEVSNGCFSWDASPEMPTLKDLNFQARQGTRVAVCGTVGSGKSSLLSCILGEVPKLSGVVRTCGTIAYVSQSAWIQSGKVQENILFGKQMDSEKYDSVLELCSLKKDLESFPSGDQTFIGERGINLSGGQKQRVQIARALYQDADIYLFDDPFSAVDAHTGSHIFKECLLGALAQKTVVYVTHQLEFLPAADLILVIKDGIIAQSGRYNEILGSGEEFMELVGAHQDALATIDTIDVPIGASEAFSSGGAASLSGSLTSAEKKDKQNVKQDDGHDQSGQLVQEEERERGRVGFWVYWKYLTLAYGGALVPFVLLAQILFEVLHIASNYWMAWAAPASKDVEPPVSMYTLIYVYVALALGSSVCTFVRALFLVPAAYKTATLLFNKMHVSIFRAPMSFFDSTPSGRILNRASTDQSLVDTSIANRMGSIAFAFIQLGGTIVVMSQVAWQVFVVFIPVIAICLWYQRYYIDTARELQRMVGICKAPIIQHFVESITGSAIIRSFGKEKQFLATNNQLMDAYSRQKFYNAGAMEWLCFRMDMLSSLTFAISLIFLINLPTGIIDPGIAGLVVTYGLNLNIMQVTLVTSMCNLENKIISVERILQYLSLPEEAPLLMSEDGLAHNWPSEGEIQLHNLHVKYAPQLPFVLKGLTVTFPGGMKTGIVGRTGSGKSTLIQALFRIMDPTVGQITVDGVDICTIGLHDLRSRLSIIPQDPTMFDGTVRHNLDPLGEYTDNQIWEALDHCQLGDEVRRKELKLDSPVVENGENWSVGQRQLVCLGRVILRRTKILVLDEATASVDTATDNLIQKTLQQHFSGATVITIAHRITSVLHSDIVLLLDNGMAVEHQTPARLLEDKSSLFSKLVAEYTMRSTRT |
| *TaABCC5* | > Traes_1BL_D21E2A0BE.1  MKQPLLDQATSSSSEATGTKSLFTDAGWFSIITFYWMGPLLDLGRKKPLDLDDVPFLDDSDSVHGVLPNFKAKIVSNSATGQFTGVTAVKLAKAIVLTTWKPILVTAVYALLSSVASYVGPYLIEYFVDYLNKSSRSTKEGYVLVLTFVAAQLIEGLSTRHLQFRSKQVGVRARSSLVAAIYQKGLALSSQSRQSNSSGEMINVVSLDAECVGDFSRSMHDLWRLPVQIVLAMLILYSTLGFCPAFAALLATALTIGGNKPLGRMEQNYQERMMSAKDVRMRAMSEILQSMRILKLQGWEMIFLSKIIELRKVEMNWLKKNVYTSAMLLSIFFSAPAFVAMVTFGVCVLMGIPLETGKVLCALATFRQLQTPIHGLPDAYSMIIQTKVSLDRICSFLCLEELPSDVVTKLPRGTTDVSIEVTNGHFSWNTSSQVPTLQDVNFRIRQGMRVAVCGTVGSGKSSLLSCILGEIPKLSGEVRTCGRISYVSQTPWIQSGKIEDNILFGTEMNRERYEKVLEACSLIKDLDILPFGDQTIIGERGINLSGGQKQRIQIARALYHDADIYLFDDPFSAVDAHTGLHLFKECLLGFLASKTVVYVTHHVEFLPSADVIMVLKDGKIIQAGDYTEILNSGKEFTELIVSHKDALSTMDMLELPSSNYESSCHLHGNGSALPIADEQTHDNNQEVLVQNGQLVQEEEREKGRVGFIVYWRYITMAHKGAFVPLILLAQIIFQSLQIGSNLWMAWAAPVSKDVNPPVNSSTMINVYVALALVTSLCVFIRSYLLVMAGCRTATMLFDKMHECIFRAPMCFFDSTPSGRILNRASTDQSAVDTQIYDLMGYLLFPAIEILGTIILMSRVAWPVFVIFVPVIIASLSYQQYYISAARELQRLTGVYRAPVMQHFAESIAGTTIIRCFDKKREFISWTGQLMDNLSRASLYNAAAMEWLCFRLDFLSSFIFGFALILLVTLPTDLIDSKTAGLAVTYGLSLNMLIGWAIMVLCALENRMISVERILQYMTIPSEPPLTISESRLDCHWPTKGEIELRNLHVKYAPHLPLVLKGVTCTFSGGMKTGIVGRTGGGKSTLIQTLFRIVDPCVGQILIDGVDISTIGLHDLRTRLSIIPQDPVMFEGTLRSNIDPLDEYNDEQIWEALDCCHLGDEVRKNELKLDSTVTENGENWSAGQRQLVCLGRVILKRRRILVLDEATSSVDPITDSLIQKTLKQQFAECTVITIAHRITSVLDSERVILLDNGEIAEHDSPARLLEDSSSLFSKLVSEYTMGSKL |
| *TaABCC6* | > Traes_2AL_2F9F6DB1A.1  MKVAICGMVGSGKSSLLSCILGEMPKLAGAVRVSGSRAYVPQTAWILSGNIRDNILFGNPYDKEKYEKIIQACALTKDLELFANGDLTEIGERGINMSGGQKQRIQIARSVYEDADIYLFDDPFSAVDAHTGAQLFKDCLMGMLKDKTILYVTHQVEFLPAADLILDGKIVQKGRFDDLLKQNIGFETIVGAHSQAIDSVINAESSSRILSTESQKLADSDDEFERENDTDDQVQGIIKQESEHDVSQGLNEKGRLTQEEEREKGGIGKTVYWAYLTAIHGGALAPVIVAAQSFFQIFQVASNYWMAWACPPTSATTPRVGLGLLLSVYLVLSIGSALCVFGRSMLVSLVGLLTAEKFFKNMLHCILRAPMSFFDSTPTGRILNRVSSDQSVLDLEIASKLGWCAFSVIQILGTIGVMSQVAWPVFAIFIPVTAICYVFQRYYIPTARELARLSQIQRAPILHHFAESLTGAASIRAYGQKDRFRKANISLVNNHSRPWFHNISAIEWLCFRLNMLSNFVFAFSLTLLVSLPEGFINPSIAGLAVTYALNLNGQLSSITWNICNTENKMISVERIMQYSRIPSEAPLIVDDHRPPNSWPKDGTINIRNLEVRYAEHLPSVLRNISCTIPGRKKVGIVGRTGSGKSTLIQALFRIVEPRVGTIEIDDVDLSKIGLHDLRGRLSIIPQDPTMFEGTVRGNLDPLNEYSDQHIWETLDKCQLGDIVRQSPKKLDSTVVENGENWSVGQRQLFCLGRILLKRSNVLVLDEATASVDSSTDAIIQQTLREEFGDCTVLTVAHRIHTVIDSDLILVFSEGRIIEYDTPSRLLEDENSEFSRLIKEYSRRSKGF  > Traes_2DL_E79ECCBC9.1  MTPLFVIGYKKPLDKNDVPDIDERDYADLLSDSFKRILADVEHRHGLSTLSIYRAMFLFIRRKAILNAVFAILCACASYVGPSLINDLVKFLGGERKNGLQKGYLLAVAFLSAKVVETIAERQWIFGAQRLGMRLGAALISHIYQKGLRLSCGARQKHSSGEIINYMSVDIQRITEVMWYTNYIWMLPIQLSLAVYVLHLNLGAGAWAGLAATLAIMTCNIPLTRLQKRLQSEIMAAKDNRMKATTEVLRSMKILKLQAWDTEYLQKLEALRREEHNWLWKSVRLSALTTFIFWGSPAFISSITFGTCILMGIPLTAGTVLSALATFRMLQDPIFTLPDLLSVFAQGKVSADRVAQYLQEEELKDDAITEVSRSDTDYDVEIDHGAFSWELETTSPTITDVNLKVKRGMKVAICGMVGSGKSSLLSCILGEMPKLAGTVRVSGSRAYVPQTAWILSGNIRDNILFGNPYDKEKYEKIIQACALTKDLELFANGDLTEIGERGINMSGGQKQRIQIARSVYEDADIYLFDDPFSAVDAHTGAQLFKDCLMGLLKDKTILYVTHQVEFLPAADLILVMQDGKIVQKGRFDDLLKQNIGFEAIVGAHSQAIDSVINAESSSRILSTESQKLADSDDEFERENDTDDQVQGIIKQESEHDVSQGVNEKGRLTQEEEREKGGIGKTVYWAYLTAVHGGALAPVIVAAQSFFQIFQVASNYWMAWACPPTSATTPRVGLGLLLSVYIMLSIGSALCVFGRSILLSLVGLLTAEKFFKNMLHCILRAPMSFFDSTPTGRILNRVSSDQSVLDLEIASKLGWCAFSVIQILGTIGVMSQVAWPVFAIFIPVTAICYAFQRYYIPTARELARLSQIQRAPILHHFAESLTGAASIRAYGQKDRFSKANISLVNNHSRPWFHNISAVEWLCFRLNMLSNFVFAFSLTLLVSLPEGFINPSIAGLAVTYALNLNGQLSSITWNICNTENKMISVERIMQYSRIPSEAPLIVDDHRPPNSWPKDGTINIRNLEVRYAEHLPSVLRNISCTIPGRKKVGIVGRTGSGKSTLIQALFRIVEPRVGTIEIDDVDLSKIGLHDLRGRLSIIPQDPTMFEGTVRGNLDPLNEYSDQHIWETLDKCQLGDIVRQSPKKLDSTVVENGENWSVGQRQLFCLGRVLLKRSNVLVLDEATASVDSSTDAIIQQTLREEFGDCTVLTVAHRIHTVIDSDLILVFSEGRIIEYDTPSRLLEDENSEFSRLIKEYSRRSKGF |
| *TaABCC7* | >Traes_2AL_9C8ACA6B9.1  MDLFTLVICSYLFAISARGKTGIISINSGITEPLLSPSAGQQTETKRTCLYGRASVLDLVTFSWMGPLFATGYKKPLDKNDVPDIDERDYADLLSDSFKRILADVERRHGLSTLSIYRAMFLFIRRKAIINAVFAILCACASYVGPSLINDLVRFLGGERKYGLKKGYILAAAFLSAKVVETVAQRQWIFGARRLGMRLRAALISHIYQKGLRLSCSARQKHTSGEIINYMSVDIQRITDVIWYTNYIWMLPIQLSLAVYVLYLNLGAGAWAGLAATLVIMACNIPLTRLQKRLQSEIMAAKDNRMKATTEVLRSMKILKLQAWDTEYLQKLEALRREEHNWLWKSVRLTAFTTFIFWGSPAFISSITFGTCILMGIPLTAGTVLSALATFRMLQDPIFTLPDLLSVFAQGKVSADRVAQYLQEEELKDDAITEVPRSDTDFDVEIDHGAFSWEPETTSPTITDVNLKVKRGMKVAICGVVGSGKSSLLSCILGEMPKLAGTVRVSGSRAYVPQTAWILSGNIRDNILFGNPYDREKYQKVIQACALTKDLELFANGDLTEIGERGINMSGGQKQRIQIARSVYEDADIYLFDDPFSAVDAHTGGQLFKDCLMGMLKDKTILYVTHQVEFLPAADLILVMQNGKIVQKGTFDDLLQQNIGFEAIVGAHSQATESVINAESSSRILSTENQKLADSDDEFERENHIDDQVEGIIKQESAHDVSQGINEKGRLTQDEEREKGGIGKTIYWAYLTAVHGGALAPIIVAAQSFFQIFQVASNYWMAWACPPTSATTPRVGLGLLFFVYIVLSIGSALCVFGRSMLVSLVGLLTAEKFFKNMLHCILRAPMSFFDSTPTGRILNRVSNDQSVLDLKMADSLGWCAFSFIQILGTIGVMSQVAWPVFVIFIPVTAICYVFQRYYIPTARELARLQQIQRAPILHHSAESLTGAASIRAYGRKDRFSKANISLVNNHLQPWFHNVSAVEWLCFRLNMLSNFVFAFSLTLLVSLPEGFINPSIAGLAVTYALNLNGQLSSITWNICNTENKMISVERIMQYSRIPSEAPLIVDDHRPPNSWPKDGTINIRNLEVRYAEHLPSVLRNISCTIPGRKKVGIVGRTGSGKSTLIQSLFRIVEPRQGTIEIDNVDLSKIGLHDLRGRLSIIPQDPTMFEGTVRGNLDPLNEYSDQHVWETLDKCQLGDIVRQSPKKLDSTVVENGENWSVGQRQLFCLGRILLKRSNVLVLDEATASVDSSTDAIIQQTLREEFGDCTVLTVAHRIHTVIDSDLILVFSEGRIIEYDTPSRLLEDKNSEFLRLIKEYSQRSKGF  >Traes_2BL_6FFD5A5E0.1 unnamed protein productMDEGGGSLHSPPLARAHEMFLLRAAALLGDSPSSIVSQYLRQWPEVYSPCFWTSTFVLIQLVFITSIVAQYLFKRIRWCRQRLKTTTPESNKHSNQEQQNADIKLGFSYQASKVCCLLILATHVPRIFFLQLQGRISGCKYPPFVLGEGIQVLSWVILSLAVFSLQKTKSAKHPLIIRAWLILSFLQSIISVIFDLRFSLSDHGYMGFAELIDLFTLVICTYLFAVSVRGKTGITSINSGITEPLLSPSAGQQAETKRTCLYGRASVLDLVTFSWMGPLFATGYKKPLDKNDVPDIDERDYADLLSHSFKRILADVERRHGLSTLSIYRAMFLFIRRKAIINAVFAILCACASYVGPSLINDLVRFLGGERKYGLKKGYILAAAFLSAKVVETVAQRQWIFGARRLGMRLRAALISHIYQKGLRLSCSARQKHTSGEIINYMSVDIQRITDVIWYTNYIWMLPIQLSLAVYVLYLNLGAGAWAGLAATLAIMACNIPLTRLQKRLQSEIMAAKDNRMKATTEVLRSMKILKLQAWDTEYLQKLEALRREEHNWLWKSVRLTAFTTFIFWGSPAFISSITFGTCILMGIPLTAGTVLSALATFRMLQDPIFTLPDLLSVFAQGKVSADRVAQYLQEEELKDDAITEVPRRDTDYDVEIDHGAFSWELETTSPTITDVNLKVKRGMKVAICGMVGSGKSSLLSCILGEMPKLAGAVRVSGSRAYVPQTAWILSGNIRDNILFGNPYDKEKYQKIIQACALTKDLELFANGDLTEIGERGINMSGGQKQRIQIARSVYEDADIYLFDDPFSAVDAHTGAQLFKDCLMGMLKDKTILYVTHQVEFLPAADLILVMQDGKIVQKGRFDDLLKQNIGFEAIVGAHSQAIDSVINAESSSRILSTESQKLADSDDEFERENDTDDQVQGIIKQESEHDVSQGVNEKGRLTQEEEREKGGIGKTVYWAYLTAVHGGALAPVIVAAQSFFQIFQVASNYWMAWACPPTSATTPRVGLGLLLSVYIMLSIGSALCVFGRSILVSLVGLLTAEKFFKNMLHCILRAPMSFFDSTPTGRILNRVSSDQSVLDLEIASKLGWCAFSVIQILGTIGVMSQVAWPVFAIFIPVTAICYAFQRYYIPTARELARLSQIQRAPILHHFAESLTGAASIRAYGQKDRFSKANISLVNNHSRPWFHNISAVEWLCFRLNMLSNFVFAFSLTLLV |
| *TaABCC8* | > Traes_5BL_D71543428.1  MSWAWMNPLIQRGYRSPLDLSDVPTLAPAHRPERMHALFLSHWPSSWARKDNNPVRHALLRCFWPLFLLNAGLALLRLTVMYVGPTLIQSFVSFTSAAERRPLWEGARLVLALLAAKAMEALCSHQYNFHCQKLGMQIRGALITALYRKGLRLSCSARQKHGLGMIVNYMAVDAQQLSDMMLQIHYLWLMPLQVGVALGLLYIYLGPPVTSALVGVFGVMAFVLLGTRRNNRYQFSLSGERDKRMKATNEMLSYMRVIKFQAWEEHFNARIGRFRRLEFGWLTRFMYSISGNIVVLWSAPTVVSALVFGTCVAVGVPLDAGLVFTATSLFKILQEPMRNFPQAMIQASQAMISLQRLDSYMTSPELDEGAVEREPAAASRDGGVAVHARDGVFTWDDEETEAGKEVLRGIDLEIRSGKLAAVVGMVGSGKSSLLGCILGEMRKVSGKVKVCGTTAYVAQTAWIQNGTIEENILFGQPMHGERYKEVIRVCCLEKDMEMMEFGDQTEIGERGINLSGGQKQRIQLARAVYQDCDIYLLDDVFSAVDAHTGSEIFKECVRGALKNKTVVLVTHQVDFLHNADIIYVMKEGTIVQSGKYDELIQRGSDFAALVAAHNSSMELVEGAAPVSDEKGETPAISRQPSRKGSGRRPSSGEAHGVVAEKASARLIKEEERASGHVSLAVYKQYMTEAWGWWGVALVVAVSVAWQGSVLASDYWLAYETDAENAASFRPALFIEVYAIIAVASVVLVSGRSFLVAFIGLQTANSFFKQILNSILHAPMSFFDTTPSGRILSRASSDQTNVDLFLPFFVWLSVSMYITVISVLVVTCQVAWPSVIAIIPLLILNLWYRGYYLATSRELTRLESITKAPVIHHFSETVQGVMTIRCFRKGDGFFQENLNRVNSSLRMDFHNNGANEWLGFRLELAGSFVLCFTALLMVTLPKSFIQPEFVGLSLSYGLSLNSVLFWAVWMSCFIENKMVSVERIKQFVNIPCEAEWRIKDCLPVANWPTRGDIEVIDLKVRYRHNTPLVLKGITLSIHGGEKIGVVGRTGSGKSTLIQALFRIVEPSEGKIIIDGVDICTLGLHDLRSRFGIIPQEPVLFEGTIRSNIDPLEEYSDVEIWQALDRCQLKEAVTSKPEKLDASVVDNGENWSVGQRQLLCLGRVMLKHSKILFMDEATASVDSQTDAVIQRIIREDFAECTIISIAHRIPTVMDCDRVLVVDAGLAKEFDRPAALIERPSLFGALVQEYANRSSDM  > Traes_5DL_7514D124D.1  MHGERYKEVIRVCCLEKDMEMMEFGDQTEIGERGINLSGGQKQRIQLARAVYQDCDIYLLDDVFSAVDAHTGSEIFKECVRGALKNKTVVLVTHQVDFLHNADIIYVMKEGTIVQSGKYDELIQRGSDFAALVAAHDSSMELVEGAAPVSDEKGETLAISRQPSRKGSGRRPSNGEASVVAEKASARLIKEEERASGHVSLAVYKQYMTEAWGWWGVALVVAVSVAWQGSVLASDYWLAYETDAENAASFRPALFIEVYAIIAVASVVLVSGRSFLVAFIGLQTANSFFKQILNSILHAPMSFFDTTPSGRILSRASSDQTNVDLFLPFFVWLSISMYITVISVLIVTCQVAWPSVIAIIPLLILNLWYRGYYLATSRELTRLESITKAPVIHHFSETVQGVMTIRCFRKGDGFFQENLNRVNSSLRMDFHNNGANEWLGFRLELAGSFVLCFTALLMVTLPKSFIQPEFVGLSLSYGLSLNSVLFWAVWMSCFIENKMVSVERIKQFVNIPCEAEWRIKDCLPVANWPTRGDIEVIDLKVRYRHNTPLVLKGITLSIHGGEKIGVVGRTGSGKSTLIQALFRIVEPSEGKIIIDGVDICTLGLHDLRSRFGIIPQEPVLFEGTIRSNIDPLEEYSDVEIWQALDRCQLKEAVTSKPEKLDASVVDNGENWSVGQRQLLCLGRVMLKHSKILFMDEATASVDSQTDAVIQRIIREDFAECTIISIAHRIPTVMDCDRVLVVDAGLAKEFDRPAALIERPSLFGALVQEYANRSSDM |
| *TaABCC9* | >Traes_2BS_C52A9FD78.1  MYIGPSLVDRFVQFVRRGGDMTEGLHLVAILLAGKAAETLASHHYEFQGQKLGMRIHAALLAVVYRKSLRLSTGARRAHGAGTIVNYMEVDAEEVSNVTHELHNLWLMPLQIAVALALLYTHLGPSVLTAVAAIAVVTVVVALANRRNMEYQFKFLGKRDERMKTITELLSYMRVIKLQAWEETFGSKISELREAELGWLAKSMYFMCANTIVLWSGPLAMTVLVFGTCVLTGVRLDAGKVFTATAFFRMLDGPMQSFPEAIAAVTQATVSLGRLDRYLLDAELDATTVEHVLDADTGLDRVVVEVHDGMFAWDVRGKKDNEKEEEENDDSDGEEDERIVEEAPALETVLKGINMKVRKGELAAVVGMVGSGKSSLLSCIMGEMDKVSGKVTVCGSTAYVAQTAWIQNGTIQENILFGQPMDAERYKEVTRSCCLQKDLEMMEFGDQTEIGERGINLSGGQKQRIQLARAVYQNCDVYLLDDVFSAVDAHTGSYIFKECLRGMLKGKTILLVTHQVDFLHNVDNIFVMKDGMIAQSGKFDELLEAGSGFSALVAAHDSSMELVEQSRQVEKTEHSQPAVVRIPSLRSRSIGKGEKVIVSPEIEAATSKIIQEEERESGQVSWRVYKLYMTEAWGWWGVVGIFGLSLVWQASDMASDYWLSYETSGGTPFNPSLFIGVYVAIAGVSMVLQVIKTFLETVMGLQTAQIFFRKMFDSILHAPMSFFDTTPSGRILSRASSDQTTIDVVLAFFVGLTISMYISVLSTIVVTCQVAWPSVIAVIPLLLLNIWYRNRYLATSRELTRLEGVTKAPVIDHFTETVVGATTIRCFKKENEFFQENLDKINSSLRMYFHNYAANEWLGFRLELIGTLVLSITAFLMISLPSNFIKKEFVGMSLSYGLSLNSLVYFAISISCMLENDMVAVERVNQFSTLPSEAAWRKEDHLPPNWPTHGDIDISDLKVRYRPNTPLILKGVNVSIRGGEKIGVVGRTGSGKSTLIQALFRLVEPAEGKMIIDGVDLCALGLHDLRSRFGIIPQEPVLFEGTIRSNIDPVGQYSDAEIWLALERCQLKDVVASKAEKLDALVADSGENWSVGQRQLLCLGRVILKQNQILFMDEATASVDSQTDATIQKITREQFSSCTIISIAHRIPTVMDCDRVLVLDAGLVKEFDAPSRLLEQPESLFVAMVQEYADRSSNL |
| *TaABCC10* | > Traes_2DS_1E247C42B.1  MGEMHKVSGKVSVYGTTACVTQTAWVRNGTIQENILFGQPMHPETYKTVLHACCLQKDLEMMEFGDQTEIGERGINLSGGQKQRIQLARAVYQDCDIYLLDDIFSAVDAHTGSTIFKECLKGLLKNKTVLLVTHQVDFLRNVDTVFVMKNGAIIQSGVYGDLLDSCSDFLALVAAHHSSMEAPGVQGHTVQNTEYSPATVNSKPIKENSNTTAIAPSNEAGSSKLIEEEEKASGQVSWHVYKLYITQAWGWWGVLLILAISVLTEGSRMASNYWLSYETSGGTIFDISMFLGVYASIVSASVVFQFISLLFIAFLGLKSAQAFFGKMFNSILRAPMSFFDTTPSGRILSRVSADQTKIDTALLFYMGIGVSMCISVVSSIAVTCQVAWPSAIAVLPLLLLNIWYRNRYIATSRGLTRLQGVTNAPVIDHLTETILGAPTIRCLGKEDGFYQTNLDRINSNLRMSFHNYAANEWFGFRLELIGTLVLSITAFLMISLPSNFIKKEFVGMSLSYGLSLNSLVYFAISISCMLENDMVSVERVNQYSALPSEAAWAVSDCLPSANWLQRGDIDIKDLEVRYRPNTPLILKGITMSIRSGEKIGVVGRTGSGKSTLVQALFRLVEPAKGQIVIDGVDICTLGLHDLRSRFGVIPQEPALFEGTVRSNIDPIGQYSEAEIWQALECCQLKDTVAAKPEKLDALVAGMGENWSVGQRQLLCFGRVILKRSQILFMDEATASVDSQTDAAIQRIIREEFRECTVISIAHRVPTVMDSDRVVVLDAGLVKEFDAPSKLMGRPSLFGAMVQEYANRSSSEQATDG |
| *TaABCC11* | > Traes_7DL_A95F658D8.1  MIAGFYAFMRTLAIAVSPILLFAFVRYSYQEERDHRFGLSLVGCVLVIKLVESLSQRHWFFDSRRTGMRIRSALMAAIFQKQLKLSSQGRKNHSTGEIVNYIAVDAYRLGDALSWFHMAWSSPLQLAFAVGTLFWALRLGAVPGLVPLIIFGFLNMPFAKLLQGYQAKFMVAQDDRLRSTSEVLNSMKIIKLQSWEEKFRSMVESLRDAEFIWLRETQMKKAYGAVMYWMSPTVVSAVMYTATAILGSAPLNASTLFTVLATLRVMAEPVRFLPEILTMMIQYKVSLDRIEKFLIEEEIKEGAERAPPHNSDIRVHVQDANFSWNASAADLTLRNVNLSINKGEKVAVCGAVGSGKSSLLYALLREIPRTSGSVDVFGSLAYVSQNSWIQSGTVRDNILFGKPFDKELYEKAIKSCALDKDIENFNHGDLTEIGQRGLNMSGGQKQRIQLARAVYSNADIYLLDDPFSAVDAHTAAVLFYDCVMTALSKKTVVLVTHQVEFLTETNRILVMEGGQVKQQGKYADLLESGTAFEKLVSAHQSSITALDTTSQENQVQGQQVLDGGIMPSALLATRQASEIEVSTRGPSVAQLTEEEEKGIGNLGWKPYKDYVEVSKGILPLCGMVTAQVLFTVFQIMSTYWLAVAIQINVSNALLVGAYSGIAIFSCCFAYLRSLFAATLGLKASKAFFTGLMDSVFKAPMSFFDSTPIGRILTRASSDLSILDFDIPYSMAFVVTGGIEVVTTVLVMGTVTWQVLVVAIPVAISMVYVQRYYVDSARELVRINGTTKAPVMNYASESILGVVTIRAFAATDRFIHNNLQLIDNDATMFFHTVAAQEWILIRVEALQSLTIFTSSLFLILVPPGVISPGFAGLCLSYALSLTAAQVFLTRYYSYLENYIISVERIKQYMHLPSEPPTIIPDNRPPISWPQEGRIDLQDLKIKYRRNTPLVLKGITCTFPAGNRIGVVGRTGSGKSTLISSLFRLVDPVGGRILIDNLDICSIGLKDLRTKLSIIPQEPTLFRGTVRNNLDPLGLHSDDEIWEALEKCQLKRSISSTAALLDTVVSDDGDNWSVGQRQLFCLGRVLLRRNKILVLDEATASIDSATDAILQGVIRQQFTSCTVITIAHRVPTVTDSDRVMVLSYGKLLEYDTPVKLLEDKQSAFAKLVAEYWANCKRNST |
| *TaABCC12* | > Traes_7DS_B65B5CD47.1  MLKILLEIPHLQYTLTEMKAMAYVTEIVSFSTSITFGLFLIVITAVRKLCNKREVNCIEAPLIPNNENSEAENANLVNKQHNVWELLTFKSVNPMMDIGIRRQLDFTDLLELPAELRTVSCYDKLLSSWTAEHQKYHADSSLLRAMFYAYGWSYLRLGILKVVNDSISFVSPLLLNKFIKLIQEGSVGMDGYIIAISLGLTSIIKSFLDTQYSFRLAKLKLMLRSSMMGIVYRKCLCLSLSERSRFSEGEIQTFMSVDVDRTVNICNNLHDAWSFPLQIGLALYLLYTQVNYAFLSGLAITTILIPVNKWISTRIATATQKMMKQKDERISCAGELLAHIRTVKMYSWDKLFTQRLNKRRELEVKHLATRKYLDAWCVYFWATTPTLFSLFTFSIFAIMGHSLDAATVFTCVALFNTLISPLNSLPWVINGMIDSVISSRRLHNYLSTPEHCSSKLTISSDIVKDDFNRNTETIYDPTAVMIRNLCCSWSSTSTVEPQIILRDISLQLQKGLFIAIVGEVGSGKSSLLNSIIGEMSVISGSINSCGSIAYVPQVPWILSGSLRDNILLGKGFDTRRYEEVIQACTLDVDISTMIGGDMSHIGEKGLNLSGGQRARLALARALYHDSDVYLFDDILSAVDSQVASWILEKAIMGPQMKRKTRLLSTHNLQAIYAADMIVVMANGFVKWFGTLDSFLATPYSKISNPDSSSAVSATSSEKNKGPSTFEFNTKDLLDNGSVVDQEEQRDQTEAEGRKEGMVEIIVYKKYATLAGWSMVFLIFVSAFLMQASRNGNDLWLTYWVDSSSGTNNTRFYLTILAGFGIINSLFTLGRAFSFAYGGLCAAIQIHADLLGSLIGAPVYFFDQNPSGRILNRLSSDLYTVDDSLPFILNIFVANFFGLLGTLVVLCYSQVTFVLILVPLWLIYSKVQFYYRSTSREVRRLDSVARSPIYSSFTETLDGSSTIRAFQKEVFFLERFIQQLTLYQKTSYSELTASLWLSLRLQLLAGLIILFIAVISVVGFHSNSPVKFGTPGLVGLALSYAAPVVSLLNSFLTTFTETEKEMISVERVVEYVGIPQEELQGSESPDRSWPTEGKIEFEHVTLRYKADLPPALNEISFHIESGMQVGIIGRTGAGKSSILNALFRLTPICNGRILVDGFDVAKVAVRDLRGHFAVVPQSPFLFDGSLRENLDPFGITTDIRIWEALEKCHMKAEIESIGGLDIHVKESGGSFSVGQRQLLCLARAILKSSKVLCLDECTANVDNQTAFLLQNTISAECKGMTVLTIAHRISTVMKMDNILVLDQGKLVEEGNPEVLMNDNRSRFSRFAKASV |
| *TaABCC13* | >Traes_4BL_7091749BF.1  MDRPRYKRVLEACSLKKDLQLLQYGDQTIIGDRGINLSGGQKQRVQLARALYQDADIYLLDDPFSAVDAHTGSDLFKDYILGALASKTVIYVTHQVEFLPAADLILVLKDGHITQAGKYDDLLQAGTDFNALVSAHNEAIETMDFGEDSDGDIAPSVPNKRLTPSVSNIDNLKNKVSENGKSSNTRGIKDKKKSEERKKKRTVQEEERERGRVSLNVYLTYMGEAYKGSLIPLIVLAQTLFQVLQIASNWWMAWANPQTEGDAPKTSSVVLLVVYMCLAFGSSLFVFVRSLLVATFGLAAAQKLFIKMLRCVFRAPMSFFDTTPSGRILNRVSVDQSVVDLDIAFRLGGFASTTIQLLGIVAVMSKVTWQVLFLIVPMAMACMWMQRYYIASSRELTRILSVQKSPVIHLFSESIAGAATIRGFDQEKRFMKRNLYLLDCFARPLFSSLAAIEWLCLRMELLSTFVFAFCMAILVSFPPGTIEPSMAGLAVTYGLNLNARMSRWILSFCKLENRIISVERIYQYCKIPSEAPLIIENCRPPASWPENGNIQLIDLKVRYKDDLPFVLHGVSCIFPGGKKIGIVGRTGSGKSTLIQALFRLIEPTGGKIIIDDIDVSAIGLHDLRSRLSIIPQDPTLFEGTIRMNLDPLEERSDQEIWEALEKCQLGEVIRSKEEKLDSPVLENGDNWSVGQRQLIALGRALLKQARILVLDEATASVDTATDNLIQKIIRSEFRDCTVCTIAHRIPTVIDSDLVMVLSDGKIAEFDTPQRLVEDKSSMFMQLVSEYSTRASCI  > Traes_4DL_B138DF6FC.1  MAAKDERMRKTAECLKSMRILKLQAWEDRYRIMLEEMRNVECRWLKWALYSQAAVTFVFWSSPIFVSVITFGTCILLGGELTAGGVLSALATFRILQEPLRNFPDLISMIAQTRVSLDRLSHFLRQEELPDDATISVPQGSTDKAIDIKDGSFSWNPSCSTPTLSHIQLSVVRGMRVAVCGVIGSGKSSLLSSILGEIPRLSGQVRVSGTAAYVSQTAWIQSGNIEENVLFGTPMDRPRYKRVLEACSLKKDLQLLQYGDQTIIGDRGINLSGGQKQRVQLARALYQDADIYLLDDPFSAVDAHTGSDLFKDYILGALASKTVIYVTHQVEFLPAADLILVLKDGHITQAGKYDDLLQAGTDFNALVSAHNEAIETMDFGEDSDGDIAPSVPNKRLTPSVSNIDNLKNKVSENGKSSNTRGIKDKKKSEERKKKRTVQEEERERGRVSLNVYLTYMGEAYKGSLIPLIVLAQTLFQVLQIASNWWMAWANPQTEGDAPKTSSVVLLVVYMCLAFGSSLFVFVRSLLVATFGLAAAQKLFIKMLRCVFRAPMSFFDTTPSGRILNRVSVDQSVVDLDIAFRLGGFASTTIQLLGIVAVMSKVTWQVLFLIVPMAMACMWMQRYYIASSRELTRILSVQKSPVIHLFSESIAGAATIRGFGQEKRFMKRNLYLLDCFARPLFSSLAAIEWLCLRMELLSTFVFAFCMAILVSFPPGTIEPSMAGLAVTYGLNLNARMSRWILSFCKLENRIISVERIYQYCKIPSEAPLIIENCRPPSSWPENGNIELIDLKVRYKDDLPFVLHGVSCIFPGGKKIGIVGRTGSGKSTLIQALFRLIEPSGGKIIIDNIDVSAIGLHDLRSRLSIIPQDPTLFEGTIRMNLDPLEERSDQEIWEALEKCQLGEVIRSKEEKLDSPVLENGDNWSVGQRQLIALGRALLKQARILVLDEATASVDTATDNLIQKIIRSEFRDCTVCTIAHRIPTVIDSDLVMVLSDGKIAEFDTPQRLVEDKSSMFMQLVSEYSTRASCI  > Traes_5AL_FA71552A8.1  MRILKLQAWEDRYRIMLEEMRNVECRWLKWALYSQAAVTFVFWSSPIFVSVITFGTCILLGGELTAGGVLSALATFRILQEPLRNFPDLISMIAQTRVSLDRLSHFLRQEELPDDATISVPQGSTDKAIDIRDGSFSWNPSCSNPTLSDIQLSVVRGMRVAVCGVIGSGKSSLLSSILGEIPKLSGQVRISGTAAYVSQTAWIQSGNIEENVLFGTPMDRPRYKRVLEACSLKKDLQLLQYGDQTIIGDRGINLSGGQKQRVQLARALYQDADIYLLDDPFSAVDAHTGSDLFKDYILGALASKTVIYVTHQVEFLPAADLILVLKDGHITQAGKYDDLLQAGTDFNALVSAHNEAIETMDFGEDSDGDIAPSVPNKRLTPSVSNIDNLKNKVSENGKSSNTRGIKDKKKSEERKKKRTVQEEERERGRVSLNVYLTYMGEAYKGSLIPLIVLAQTLFQVLQIASNWWMAWANPQTEGDAPKTSSVVLLVVYMCLAFGSSLFVFVRSLLVATFGLAAAQKLFIKMLRCVFRAPMSFFDTTPSGRILNRVSVDQSVVDLDIAFRLGGFASTTIQLLGIVAVMSKVTWQVLFLIVPMAMACMWMQRYYIASSRELTRILSVQKSPVIHLFSESIAGAATIRGFGQEKRFMKRNLYLLDCFARPLFSSLAAIEWLCLRMELLSTFVFAFCMAILVSFPPGTIEPSMAGLAVTYGLNLNARMSRWILSFCKLENRIISVERIYQYCKIPSEAPLIIENCRPPSSWPENGNIELIDLKVRYKDDLPFVLHGVSCIFPGGKKIGIVGRTGSGKSTLIQALFRLIEPAGGKIIIDNIDASAIGLHDLRSRLSIIPQDPTLFEGTIRMNLDPLEERSDQEIWEALEKCQLGEVIRSKEEKLDSPVLENGDNWSVGQRQLIALGRALLKQARILVLDEATASVDTATDNLIQKIIRSEFRDCTVCTIAHRIPTVIDSDLVMVLSDGKIAEFDTPQRLLEDKSSMFMQLVSEYSTRASCI |
| *TaABCC14* | > Traes_3B_74B89A214.1  MRVVSEPMRMLPEVMSVMIQVKVSLDRIGKFLTEDEFQDDAVDRTPASDKSLDMHNGVFSWEPSKGTATLKDINITATRGQKIAVCGPVGAGKSSLLCATLGEIPRMSGSVAVSGSVAYVSQTSWIQSGTVRDNILFGKPMRSSEYERALKCCALDKDMENFPHGDLTEIGQRGLNMSGGQKQRIQLARAVYNDADVYLLDDPFSAVDAHTAATLFNDCVMAALEDKTVILVTHQVEFLSKVDRILVMEKGEITQEGTYEELLQFGTAFEQLVNAHQDSKTTLDSNVSKEGAMIQYQQPILPQQGSDAEISTGNLPSVQLTQEEERELGGAGLKTYKDYVSVSKGWFLLVLIILTQCVFVALQYLATYWLAATIQSRRFSVGIVVGVYAVMTTTSCLFAYVRSLVAAHFGLKASREFFSGFMDSVFKAPMLFFDSTPTGRIMTRASSDLCILDFDIPFTMTFVISGTVEVAATVVVMIMVTWQVVLVAVPAVIGVLYIQRYYIASARELVRINGTTKAPVMNYAAESMLGVVTIRAFAATNRFIQTNLQLIDMDATMFFYTNAALEWVLLRVEAMQILVIVTSSILLVMLPAGSVAPGFLGLCLSYALTLSSAQVFLTRFYSNLENYMISVERIKQFMHLPSEPPAVISDRRPAPSWPSEGKINLENLRVKYRENAPTVLRGITCTFAAGNKIGVVGRTGSGKTTLLSALFRLIDPSGGRILIDDVDICTIGLKDLRMKLSIIPQEPTLFRGSVRSNVDPLGLYTDQDIWEALDKCQLKKTISVLPELLEAPVSDDGENWSAGQRQLFCLARVLLSRNRILVLDEATASIDSATDAILQRVIKQEFSGCTVITIAHRVPTVTDSDIVMVLSYGKLIEYDRPSRLMENEDSSFFKLVAEYWSNYK |
| *TaABCC15* | > Traes_7AS_8CC30284A.1  MMNLCGGPVCSNQDVLSCAFKEVFDSSTCTNHLAATGIALLLVLALSLQLVIKIPKSGASAQGLVAVGSPLQLAAVVFSGILGLVYLGLGLSMLGSIFSQDASVYLPHWWLVTLSQGFSLVLSSFAFSVRPWFLGASFVPVWSILVTLYAAFICCSSVVGIVADKAVTIKACLDVLSLPAAFLFLLYGVRRSHDEDDYQATGNALYKPLNTEADDQIADSDTQVTSFAKAGFFSKMSFWWLNHLMKMGYKKPLEDKDMPLLQTTDRAHNQYLMFLEKLNSKQSQSHATPSILWTIVSCHKREIIVSGFFALLKVLTLSTGPLLLKAFINVSVGKGTFKYEGFVLAATMFVCKCCESLSQRQWFFRTRRLGLQVRSFLSAAIYKKQQKLSNSAKMKHSSGQIMNYVTVDAYRIGEFPYWFHQTWTTSLQLCIALAILYNAVGAAAVSSLAVIIITVIGNAPVAKLQHKFQSKLMEAQDVRLKAMSESLVHMKILKLYSWEGHFKKVIEGLREVEYKWLSAFLLRRAYNSFLFWSSPVLVSAATFLTCYLFKIPLDASNVFTTVATLRLVQDPVRTIPDVIAVLIQAKVGFTRISKFLDAPELNGQVRKKYRAGIDYPIAMNSCSFSWDENPSKPTLNNINLVVKAGEKVAICGEVGSGKSTLLASVLGEVPKTEGTIEVCGKIAYVSQTAWIQTGTVQDNILFGSLMDRQIYQETIERCSLVKDLEMLPFGDRTQIGERGVNLSGGQKQRVQLARALYQNADIYLLDDPFSAVDAHTATSLFNDYVMGVLSDKTVLLVTHQVDFLPVFDSILLMSDGEVIRSAPYQDLLADCQEFKYLVNAHKDTVGVQDPNSAPHGAKEIPTKETDGIHVNRYIESVGPSPVDQLIKKEERESGDTGLKPYMLYLRQNKGFLYASLSVMSHIVFLAGQISQNSWMAANVQNPHVSTLKLISVYVGIGVCTMIFVLSRCLFVVVLGVQTSRSLFTQLLNSLFRAPMSFFDSTPQGRILSRVSSDLSIVDLDIPFAFMFSLSSCLNAYSNVGVLAVVVWQVLFVALPMIVLVIQLQRYYLASAKELMRINGTTKSALANHLGESISGAITIRAFEEEDRFFAKNLELVDKNAGPYFFNFAATEWLIERLEIMGAVVLSSSAFVMALLPAGTFSPGFIGMALSYGLSLNNSFVNTIQKQCDLANKIISVERVNQYMDIPSEAPEVIEENRPAPDWPQVGSVELKDLKIRYREDAPLVLHGISCKFQGRDKIGIVGRTGSGKTTLIGALFRLVEPAEGKIIIDSVDISTIGLHDLRSRLGIIPQDPTLFQGTVRYNLDPLGQFSDQQIWEVLDKCQLLEAVQEKKQGLDSLVAEDGSNWSMGQRQLFCLGRTLLKRCRILVLDEATASIDNTTDAVLQKTIRTEFKHCTVITVAHRIPTVMDCDMVLAMSDGKVAEYDKPSKLMETEGSLFRELVNEYWSYTSNGNI  >Traes_7BS_BB0FECDBE.1  MFSLSSSLNAYSNVGVLAVVVWQVLFVALPMIVLVIQLQRYYLASAKELMRINGTTKSALANHLGESISGAITIRAFEEEDRFFAKNLELVDKNAGPYFFNFAATEWLIERLEIMGAVVLSSSAFVMALLPAGTFSPGFIGMALSYGLSLNNSFVNTIQKQCDLANKIISVERVNQYMDIPSEAPEVIEENRPAPDWPQVGSVELKDLKIRYREDAPLVLHGITCKFQGRDKIGIVGRTGSGKTTLIGALFRLVEPAEGKIIIDSVDISTIGLHDLRSRLGIIPQDPTLFQGTVRYNLDPLGQFSDQQIWEVLDKCQLLEAVQEKKQGLDSLVAEDGSNWSMGQRQLFCLGRTLLKRCRILVLDEATASIDNTTDAVLQKTIRTEFKHCTVITVAHRIPTVMDCDMVLAMSDGKVAEYDKPSKLMETEGSLFRELVNEYWSYTSNGNI |
| *TaABCC16* | >lcl\|Traes_7AS_8CC30284A.2 unnamed protein product  MGSLTSSWMMNLCGGPVCSNQDVLSCAFKEVFDSSTCTNHLAATGIALLLVLALSLQLVIKIPKSGASAQGLVAVGSPLQLAAVVFSGILGLVYLGLGLSMLGSIFSQDASVYLPHWWLVTLSQGFSLVLSSFAFSVRPWFLGASFVPVWSILVTLYAAFICCSSVVGIVADKAVTIKACLDVLSLPAAFLFLLYGVRRSHDEDDYQATGNALYKPLNTEADDQIADSDTQVTSFAKAGFFSKMSFWWLNHLMKMGYKKPLEDKDMPLLQTTDRAHNQYLMFLEKLNSKQSQSHATPSILWTIVSCHKREIIVSGFFALLKVLTLSTGPLLLKAFINVSVGKGTFKYEGFVLAATMFVCKCCESLSQRQWFFRTRRLGLQVRSFLSAAIYKKQQKLSNSAKMKHSSGQIMNYVTVDAYRIGEFPYWFHQTWTTSLQLCIALAILYNAVGAAAVSSLAVIIITVIGNAPVAKLQHKFQSKLMEAQDVRLKAMSESLVHMKILKLYSWEGHFKKVIEGLREVEYKWLSAFLLRRAYNSFLFWSSPVLVSAATFLTCYLFKIPLDASNVFTTVATLRLVQDPVRTIPDVIAVLIQAKVGFTRISKFLDAPELNGQVRKKYRAGIDYPIAMNSCSFSWDENPSKPTLNNINLVVKAGEKVAICGEVGSGKSTLLASVLGEVPKTEGTIEVCGKIAYVSQTAWIQTGTVQDNILFGSLMDRQIYQETIERCSLVKDLEMLPFGDRTQIGERGVNLSGGQKQRVQLARALYQNADIYLLDDPFSAVDAHTATSLFNDYVMGVLSDKTVLLVTHQVDFLPVFDSILLMSDGEVIRSAPYQDLLADCQEFKYLVNAHKDTVGVQDPNSAPHGAKEIPTKETDGIHVNRYIESVGPSPVDQLIKKEERESGDTGLKPYMLYLRQNKGFLYASLSVMSHIVFLAGQISQNSWMAANVQNPHVSTLKLISVYVGIGVCTMIFVLSRCLFVVVLGVQTSRSLFTQLLNSLFRAPMSFFDSTPQGRILSRVSSDLSIVDLDIPFAFMFSLSSCLNAYSNVGVLAVVVWQVLFVALPMIVLVIQLQRYYLASAKELMRINGTTKSALANHLGESISGAITIRAFEEEDRFFAKNLELVDKNAGPYFFNFAATEWLIERLEIMGAVVLSSSAFVMALLPAGTFSPGFIGMALSYGLSLNNSFVNTIQKQCDLANKIISVERVNQYMDIPSEAPEVIEENRPAPDWPQVGSVELKDLKIRYREDAPLVLHGISCKFQGRDKIGIVGRTGSGKTTLIGALFRLVEPAEGKIIIDSVDISTIGLHDLRSRLGIIPQDPTLFQGTVRYNLDPLGQFSDQQIWEVLDKCQLLEAVQEKKQGLDSLVAEDGSNWSMGQRQLFCLGRTLLKRCRILVLDEATASIDNTTDAVLQKTIRTEFKHCTVITVAHRIPTVMDCDMVLAMSDGKVAEYDKPSKLMETEGSLFRELVNEYWSYTSNGNI |
| *TaABCC17* | > Traes_7AS_86392D8DF.1  MGSFTGSSWVMSLCGSPICSHQDVASCTFKEIFDASTCMNHLAATGIVALLLFALALQLFVKIPKSRASARQLVTLSSPLHLSAVVFSGTLGLVYLGLGLWMLGSGFSQDDSAYLPHWWLVTVCQGLNLILASFAFSIRPRFLGAAFVRFWPVLLTVYAAFICSSSAVDIVAEKALTVKGCLDILSLPGAVLMLIYGIRHSHHEEGHGGSGNGLYKPLNTEADSEVADSDSQVTPFAKAGFFSRMSFWWLNPLMKMGYEKPLEDNDMPLLGATDRAQNQYLMFMEKLNREKQSPSHATPSFFWTIVSCHKRAILVSGFCALLKVLTLSTGPMLLKAFINVSLGKGSFKYEGFVLAAVMFVCKFCESLSQRQWYFRTRRLGLQVRSFLSAAIYKKQQKLSNAAKMKHSSGEIMNYVTVDAYRIGEFPYWFHQTWTTSVQLCIALAILYNAVGSAMLSSLVVIIITVLCNAPLAKLQHKYQSKLMEAQDVRLKAMTESLVHMKVLKLYAWEAHFKKVIEGLREVEYKWLTAFQLRRAYNSFLFWSSPVLVSAATFLTCYLLKIPLDASNVFTFVATLRLVQDPIRQIPDVIGVVIQAKVAFTRISKFLDAPELNGQARKKYYFGIDYPIAMNSCSFSWDENPLKPTLKNINLAVKVGEKVAICGEVGSGKSTLLAAVLGEVPKTEGTIQVCGKIAYISQNAWIQTGTVQDNILFGSPMDRERYHNTLVRCSLVKDLEMLPYGDCTQIGERGVNLSGGQKQRVQLARALYQNADIYLLDDPFSAVDAHTATSLFNEYVMSALSDKTVLLVTHQVDFLPVFDSILLMSDGEVIRSAPYQDLLADCEEFKDLVNAHKDTIGVSNVNNNIPTRRSKEVSVKETDGIHTESVKPSPADQLIKKEERETGDAGVKPYMLYLCQNKGLLYFSFCIISHIIFVAGQISQNSWMAANVQNPHVSTLKLISVYIIIGVCTVFFLLSRSLAVVVLGIQTSRSLFSQLLNSLFRAPMSFFDSTPLGRVLSRVSSDLSIVDLDVPFAFVFSLGASLNAYSNLGRYYLASAKELMRINGTTKSALANHLGESIAGAITIRAFEEEDRFFAKNLDLVDKNASPYFYNFASTEWLIQRLEIMSAAVLSFSAFVMALLPQGTFSPGFVGMALSYGLSLNMSFVFSIQNQCNLANQIISVERVNQYMDIQSEAAEVVEENRPSPDWPQDGNVELRDLKIRYRKDAPLVLHGITCRFEAGNKIGIVGRTGSGKTTLIGALFRLVEPAEGKIIIDSVDISTIGLHDLRSRLGIIPQDPTLFQGTVRYNLDPLGQFSDQQIWEVLDKCQLLEAVQEKEQGLDSHVVEDGSNWSMGQRQLFCLGRALLRRCRILVLDEATASIDNATDAVLQKTIRTEFKYCTVITVAHRIPTVMDCDMVLAMSDGRVVEYDKPTKLMETEGSLFHKLVNEYWSYTSNGNI |
| *TaABCC18* | ------------------------------------- |

.
